# Supplementary material for: Functional and genetic markers of niche partitioning among enigmatic members of the human oral microbiome
Source: Genome Biol. 2020 Dec 16;21:292. doi: 10.1186/s13059-020-02195-w (PMC7739484; doi:10.1186/s13059-020-02195-w)
Supplement: Supplementary file 4 — Additional file 4. Supplementary figures S1, S2, S3, S4, S5, S6, and S7. [file 13059_2020_2195_MOESM4_ESM.pdf]

Supplementary Figures for “Functional and genetic markers of niche partitioning among enigmatic members of the human oral microbiome” by Shaiber et al.

Other supplementary files associated with this work are accessible via [doi:10.6084/m9.figshare.11634321](https://doi.org/10.6084/m9.figshare.11634321).

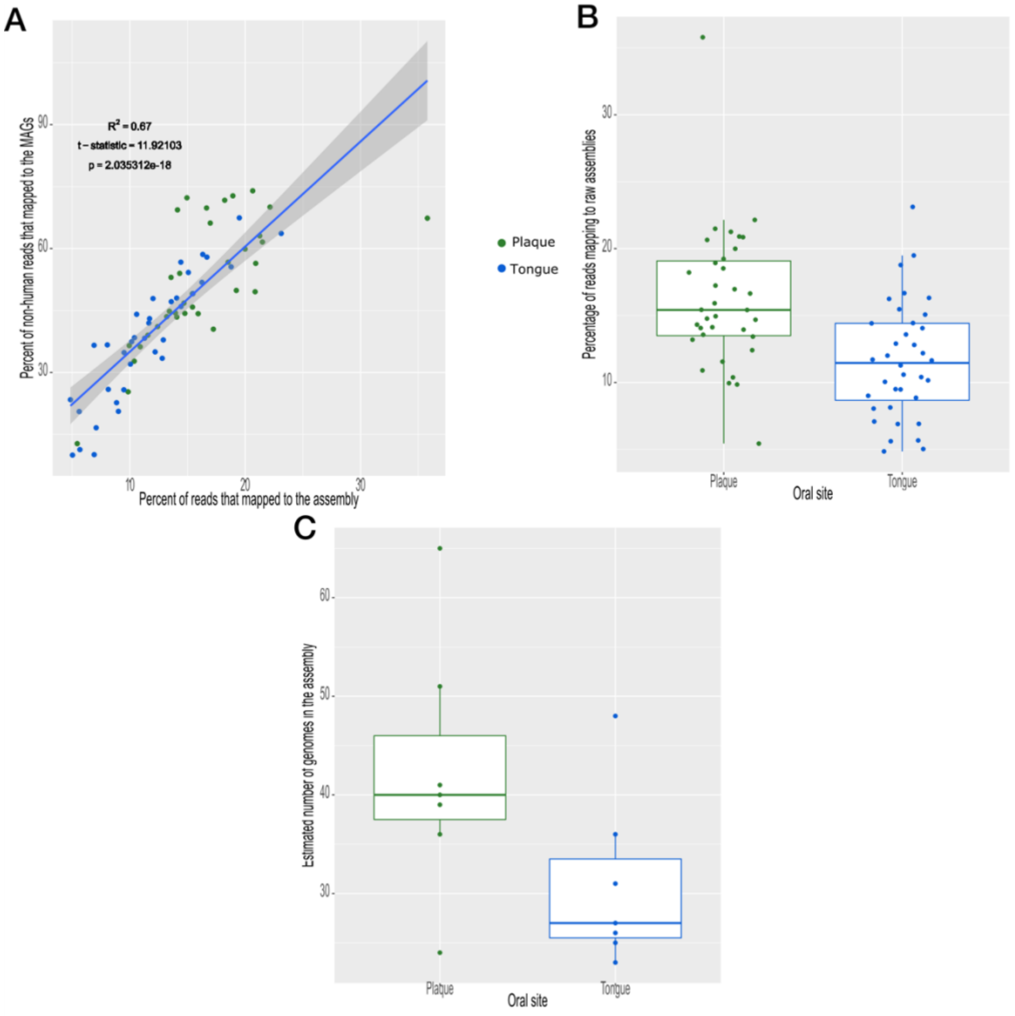

Figure S1: (A) The percent of reads that map to MAGs is correlated with the quality of the assembly. The percent of reads that mapped to the non-redundant collection of MAGs out of the total number of reads, excluding reads that mapped to the human genome is presented for each of the 35 plaque (green) and 36 tongue (blue) metagenomes as a function of the percent of reads that mapped to all contigs in the assembly. Blue curve represents a linear regression model with the grey shaded area marking the 95% confidence intervals. R-squared value and p-value for the linear regression appear above the curve. (B) A significantly higher percentage of reads map to plaque assemblies as compared to tongue assemblies. Box and whisker plots for the percentage of reads that map to the raw (i.e. without binning) assembly contigs for plaque (green) and tongue (blue) metagenomes. (C) Higher number of estimated genomes in assemblies of plaque metagenomes as compared to assemblies of tongue metagenomes. Box and whisker plots for the number of estimated genomes, based on single copy core genes, in assemblies of plaque (green) and tongue (blue) metagenomes.

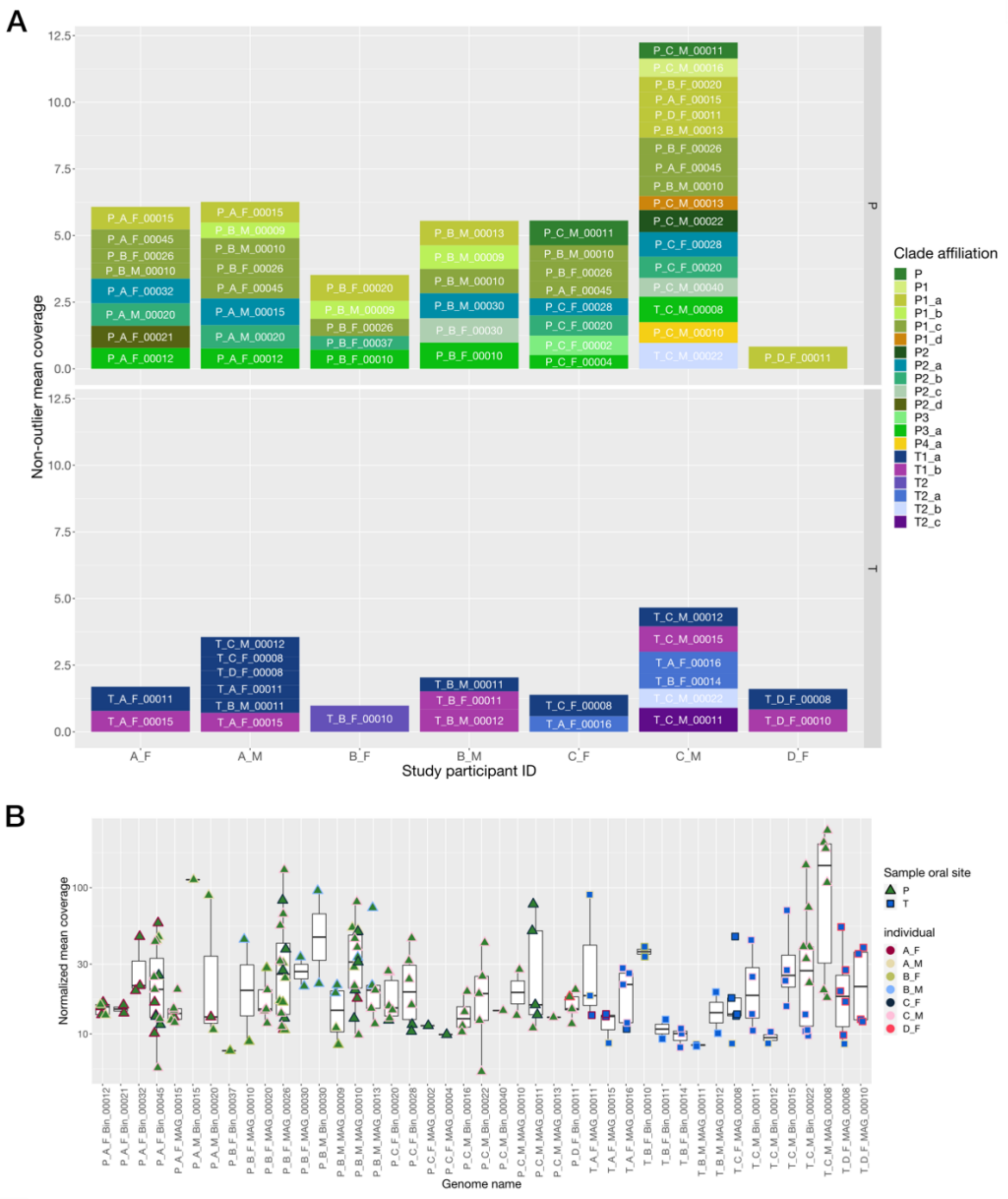

Figure S2: (A) Normalized relative abundances of TM7 population per individual for the participants of our study. For those cases in which multiple closely related populations were recovered from multiple participants, each population is detected only in the participant from which it was recovered. The exceptions are when a closely related population exists, but assembly or binning failed to recover this population. In those cases of assembly/binning failure, each of the closely related population is recovered with similar abundance. (B) Normalized relative abundances of each of our 43 TM7 MAGs in the 71 metagenomes. The shape and fill color of each dot is according to the sample type (tongue/plaque), while the stroke color is according to the participant ID from which the sample was taken.

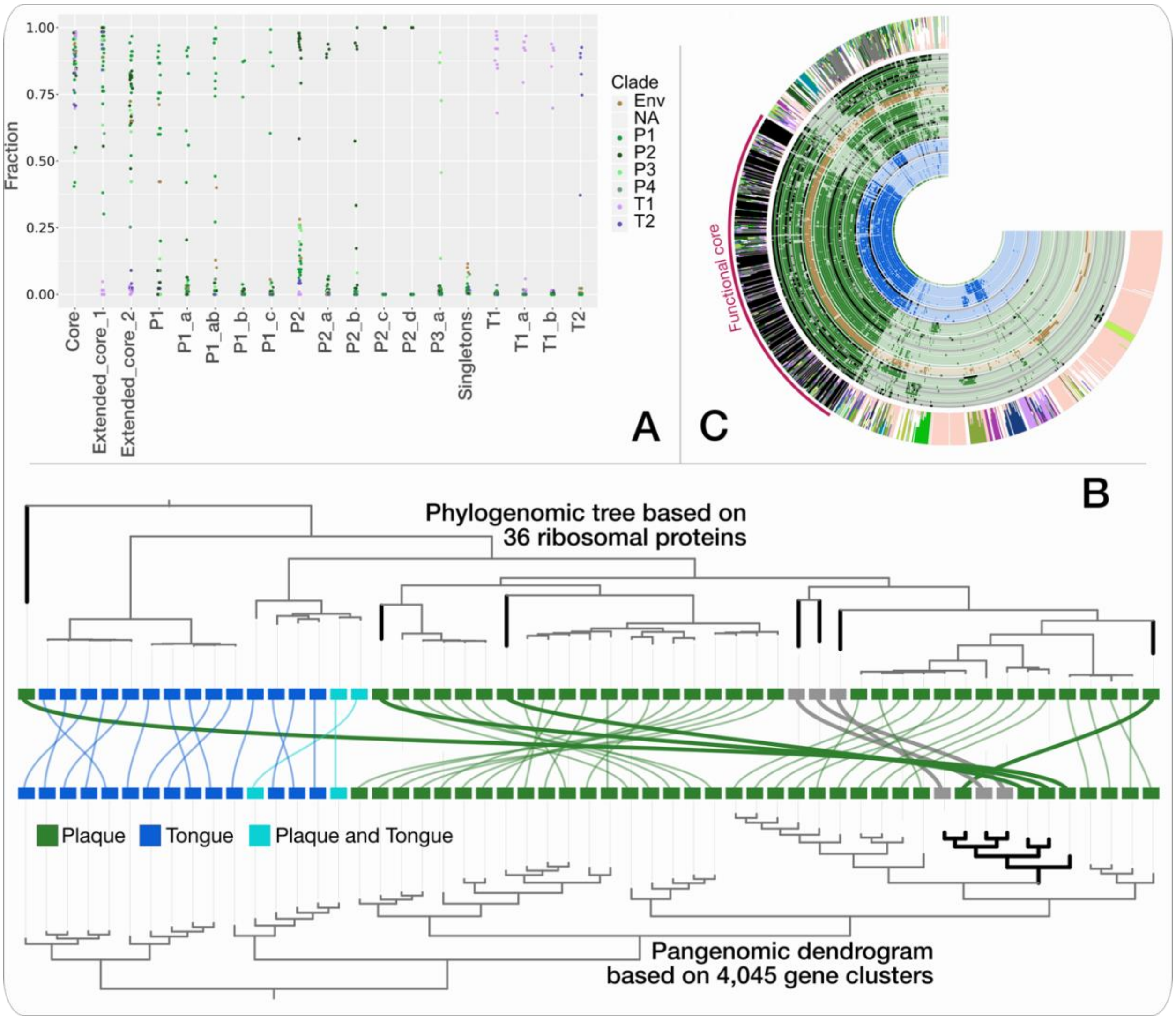

Figure S3: (A) Clade-specific groups of GCs. Data points represent the fraction of the GCs in a given group of GCs in each genome. (B) 3Organization of TM7 genomes according to the occurrence of gene clusters predicts oral site affiliation. The dendrogram at the top represents the phylogenetic organization based on ribosomal proteins, while the dendrogram on the bottom represents the hierarchical organization of genomes based on the GC frequency of occurrence across genomes using Euclidean distance and ward ordination. The information at the center of the figure shows the site affiliation of each oral TM7 in accordance with Figure 4. Branches that appear in bold black color represent environmental and plaque-associated genomes that are phylogenetically-distinct, but that are grouped together based on their gene content, and nested together with plaque-associated genomes. (C) Functional core includes mostly core GCs, but also many clade specific GCs. Each of the 970 functions are organized in the tree in the center of the figure according to their occurrence in the 55 genomes (using Euclidean distance and Ward's

method). The first 55 layers correspond to the TM7 genomes, where layers corresponding to tongue MAGs are blue, plaque MAGs are green, and previously published genomes are black. Bars in these 55 layers represent the presence of a function in the genome. The layers are ordered using the phylogenetic tree from Figure2b. The next layer includes a stacked bar representing the portion of GC bin affiliation of each gene associated with a function. The red arc in the outermost layer marks the functions that were defined as part of the core for this TM7 pangenome. Notice that while the majority of the core functions are associated with core GCs, there are many that are associated with clade-specific GCs.

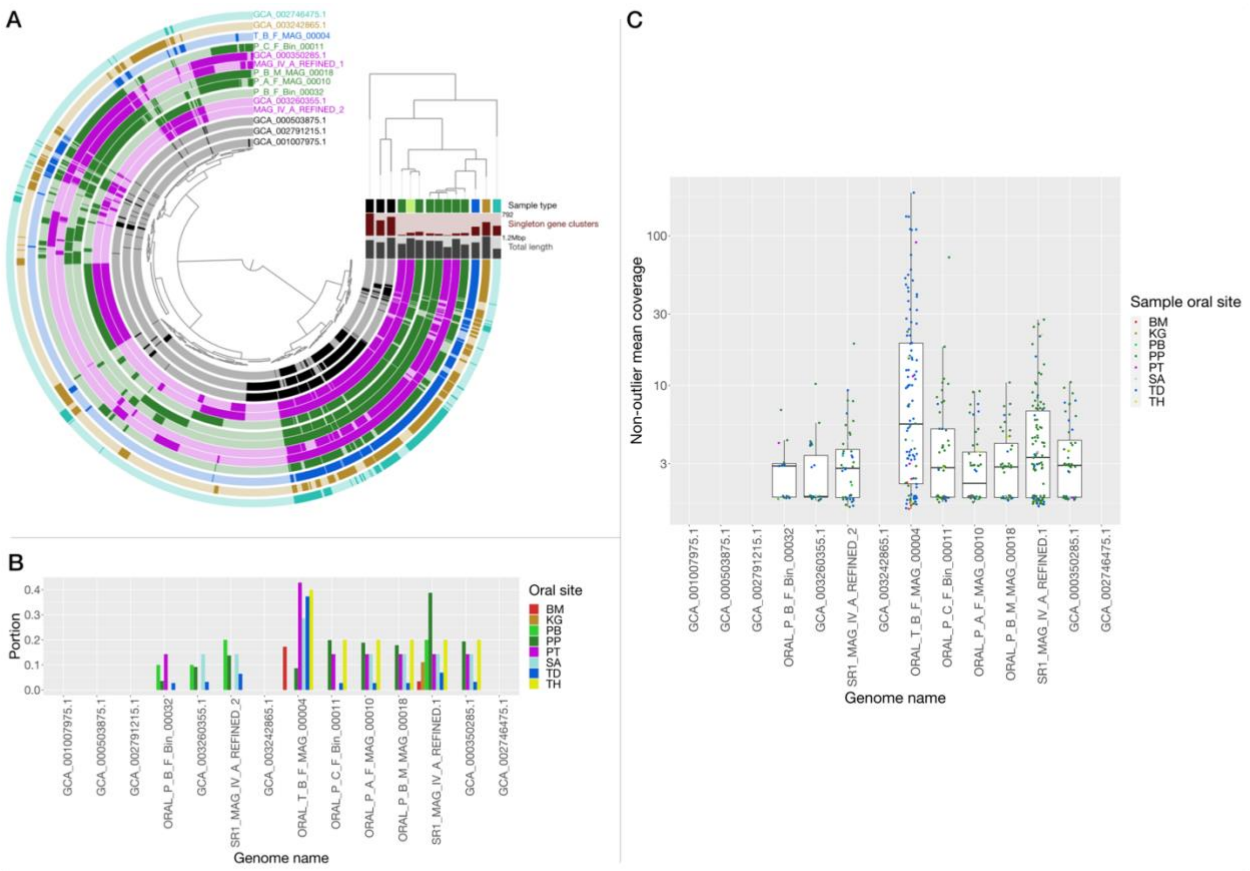

Figure S4: (A) pangenomic analysis of SR1 genomes. The dendrogram at the center of the figure organizes gene clusters (GCs) according to their occurrence across the 14 SR1 genomes. The circular layers correspond to the 14 SR1 genomes and are ordered according to their phylogenetic organization. In these circular layers, colored sections mark the presence of GCs in the corresponding genome. On the top right, the phylogenetic tree is shown and below it, the four horizontal layers correspond to (top to bottom): (1) Sample type (environmental: black, plaque: dark green, saliva: light green, canine supragingival plaque: brown, tongue: blue, dolphin gingival sulcus: cyan); (2) Number of singleton GCs; (3) Total length of the genome. (B) Detection of SR1 populations in the HMP plaque and tongue samples reveals prevalent populations and niche specificity. Barplots showing the portion of plaque (green) and tongue (blue) HMP samples in which each SR1 was detected, using a detection threshold of 0.5. (C) Normalized coverage of SR1 populations in HMP oral samples according to sample type. Boxplots showing the normalized coverages of each SR1 in plaque (green) and tongue (blue) HMP. For each genome, data is only shown for samples in which it was detected, according to the same criteria of detection used in Figure S4b.

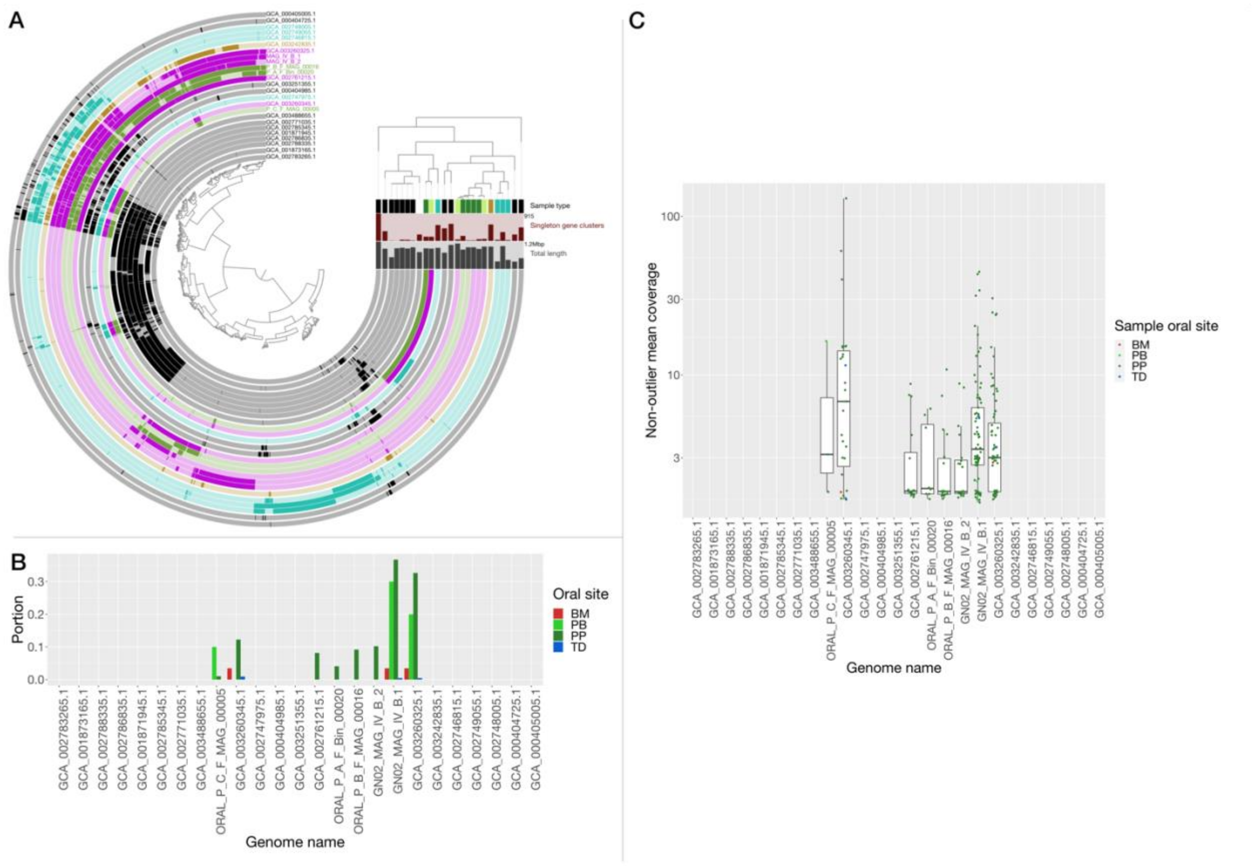

Figure S5: (A) a - pangenomic analysis of GN02 genomes. The dendrogram at the center of the figure organizes Gene clusters (GCs) according to their occurrence across the 25 SR1 genomes. The circular layers correspond to the 25 SR1 genomes and are ordered according to their phylogenetic organization. In these circular layers, colored sections mark the presence of gene-clusters in the corresponding genome. On the top right, the phylogenetic tree is shown and below it, the four horizontal layers correspond to (top to bottom): (1) Sample type (environmental: black, plaque: dark green, saliva: light green, canine supragingival plaque: brown, tongue: blue, dolphin gingival sulcus: cyan); (2) Number of singleton GCs; (3) Total length of the genome. (B) Detection of GN02 populations in the HMP plaque and tongue samples reveals the plaque specificity of oral members of this candidate phylum. Barplots showing the portion of plaque (green) and tongue (blue) HMP samples in which each GN02 was detected, using a detection threshold of 0.5. (C) Normalized coverage of GN02 populations in HMP oral samples according to sample type. Boxplots showing the normalized coverages of each GN02 in plaque (green) and tongue (blue) HMP. For each genome, data is only shown for samples in which it was detected, according to the same criteria of detection used in Fig. S5b.

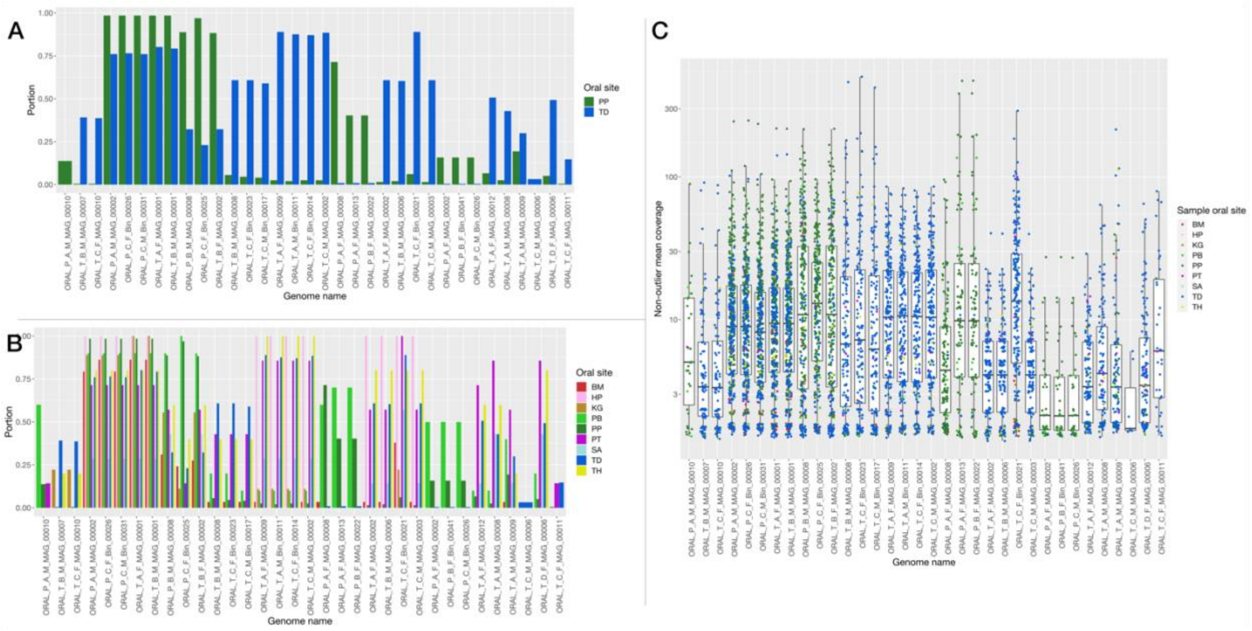

Figure S6: (A) Presence of the novel populations in HMP tongue and plaque samples. Barplots of the portion of plaque (green) and tongue (blue) samples in which each of the novel genomes occur. The presence of a population in a sample was determined according to a threshold of 0.5 detection value. (B) Presence of the novel populations in HMP oral samples by sample type. Barplots of the portion of samples in which each of the novel genomes occur, plotted by sample type for all 9 HMP sample types in which at least one novel population was detected. The presence of a population in a sample was determined according to a threshold of 0.5 detection value. (C) Normalized coverage of the novel populations in HMP oral samples according to sample type. Boxplots of the normalized coverage of the novel population. Color of data-points are according to the sample type. For each genome, data points are only shown for samples in which the genome was detected, according to the same detection threshold used in Figure S6b.

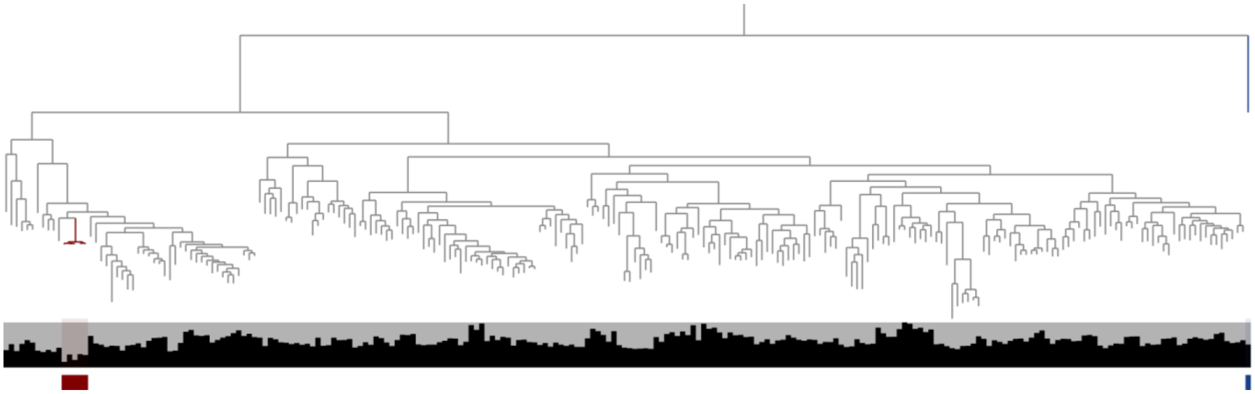

Figure S7 - Phylogenomic analysis of Flavobacteriaceae genomes indicates oral MAGs represent an unnamed species in an unnamed genus within Flavobacteriaceae. Below the dendrogram, bars indicate length of each genome (with a maximum length of 6Mbp). The 5 novel Flavobacteriaceae MAGs are indicated with red color and the Prevotella genome that was used to root the tree is indicated with blue color.
