# Supplementary material for: Functional and genetic markers of niche partitioning among enigmatic members of the human oral microbiome
Source: Genome Biol. 2020 Dec 16;21:292. doi: 10.1186/s13059-020-02195-w (PMC7739484; doi:10.1186/s13059-020-02195-w)
Supplement: Supplementary file 8 — Additional file 8. Additional discussion and supplementary figures for (A) taxonomy based on metagenome-assembled genomes, metagenomics short reads, and 16S rRNA gene amplicons; (B) Average Nucleotide Identity (ANI) of oral TM7; (C) occurrence of TM7 across additional oral sample types, other than supragingival plaque and tongue dorsum, and including samples from patients with periodontitis; (D) mobile elements and prophages in TM7 genomes; (E) novel non-CPR MAGs; (F) a novel MAG that represents a member of the Mollicutes; (G) novel Clostridiales MAGs represent prevalent tongue-associated populations; and (H) novel Bacteroidia MAGs that include a tongue-specialist and a subgingival plaque specialist. [file 13059_2020_2195_MOESM8_ESM.pdf]

Supplementary Information for "Functional and genetic markers of niche partitioning among enigmatic members of the human oral microbiome" by Shaiber et al.

All supplementary tables mentioned in this supplementary information file are accessible via doi:10.6084/m9.figshare.11634321.

(A) Taxonomy based on metagenome-assembled genomes, metagenomics short reads, and 16S rRNA gene amplicons

To investigate the taxonomic coverage of our MAGs, we compared the taxonomic composition of our samples using two additional methods: (1) metagenomic short reads using KrakenUniq (Breitwieser, Baker, and Salzberg 2018), and (2) 16S ribosomal RNA gene amplicons using and Minimum Entropy Decomposition (Eren et al. 2015) combined with GAST (Huse et al. 2008). Such comparisons are inherently very difficult for multiple reasons. For instance, while assigning taxonomy to metagenomic short reads circumvents potential challenges due to assembly and binning, it suffers from the heavy reliance on reference genomes. In parallel, quantitative estimates of the taxonomic composition through 16S rRNA gene amplicons can suffer from primer biases and rRNA operon copy number variance across organisms.

For our data, KrakenUniq estimated 441 genera to be present in at least one sample with more than zero abundance (Supplementary Table 4f). This number differed from GAST (Supplementary Table 5e) and MAGs (Supplementary Table 2f), which estimated 40 and 37 distinct genera, respectively. For a qualitative comparison we included the 15 most abundant genera according to each method, which amounted to a list of 19 genera. To this list we have manually added TM7 since it has become a primary focus of our work.

The final list of 20 top genera included *Actinomyces*, *Aggregatibacter, Campylobacter, Capnocytophaga, Corynebacterium, Derxia, Fusobacterium, Gemella, Granulicatella, Haemophilus, Leptotrichia, Neisseria, Porphyromonas, Prevotella, Pseudomonas, Rothia, Streptococcus, Streptomyces*, TM7, *Veillonella* (here we considered TM7 as a "genus" for the sake of this analysis, despite the fact that it includes multiple genera). A comparison of the relative abundance estimations by each method suggested similar trends for most of these 20 taxa, but also revealed further discrepancies across methods (Figure SI1). For instance, *Derxia* was completely absent from both KrakenUniq and MAGs, and *Gemella* and *Granulicatella* were completely absent from KrakenUniq. On the other hand, *Pseudomonas* and *Streptomyces* appear in the top 15 abundant genera of the KrakenUniq results but were completely absent from the MAGs and 16S rRNA gene amplicons. Lastly, TM7 was completely absent from the 16S rRNA amplicons, despite being amongst the top abundant genera according to MAGs.

It is difficult to reconcile these differences, which likely influence each microbial branch differently for each method. While 16S rRNA amplicons allow the taxonomic assignment of each sequenced amplicon (to various levels of resolution), it suffers from primer biases for specific taxa (Eloe-Fadrosh et al. 2016). While the study of metagenomes does not suffer from these primer biases, the ability to assign taxonomy to every sequenced read is limited by the reference database, leaving many reads either unidentified, or worse, wrongly classified (Escobar-Zepeda et al. 2018). While MAGs allow a confident taxonomic assignment (to known taxa), normalizing coverages to estimate relative abundance is challenging, especially when it is required to account for many unassigned reads. In addition, the occurrence of populations that undergo genomic reorganizations, and the occurrence of populations with large within-population variability, limits the ability to assemble short reads into large contigs and hence our ability to generate high quality MAGs. In conclusion, we could examine trends of particular taxa as these are revealed by a particular method, but none of these methods is likely to inform us of actual relative abundances. With these limitations in mind, our data shows that while the abundance profiles at the genus level are similar for the majority of the abundant genera, there are specific taxa for which there are major differences, such as *Actinomyces*, *Rothia*, and *Fusobacterium* (Figures SI1,2,3).

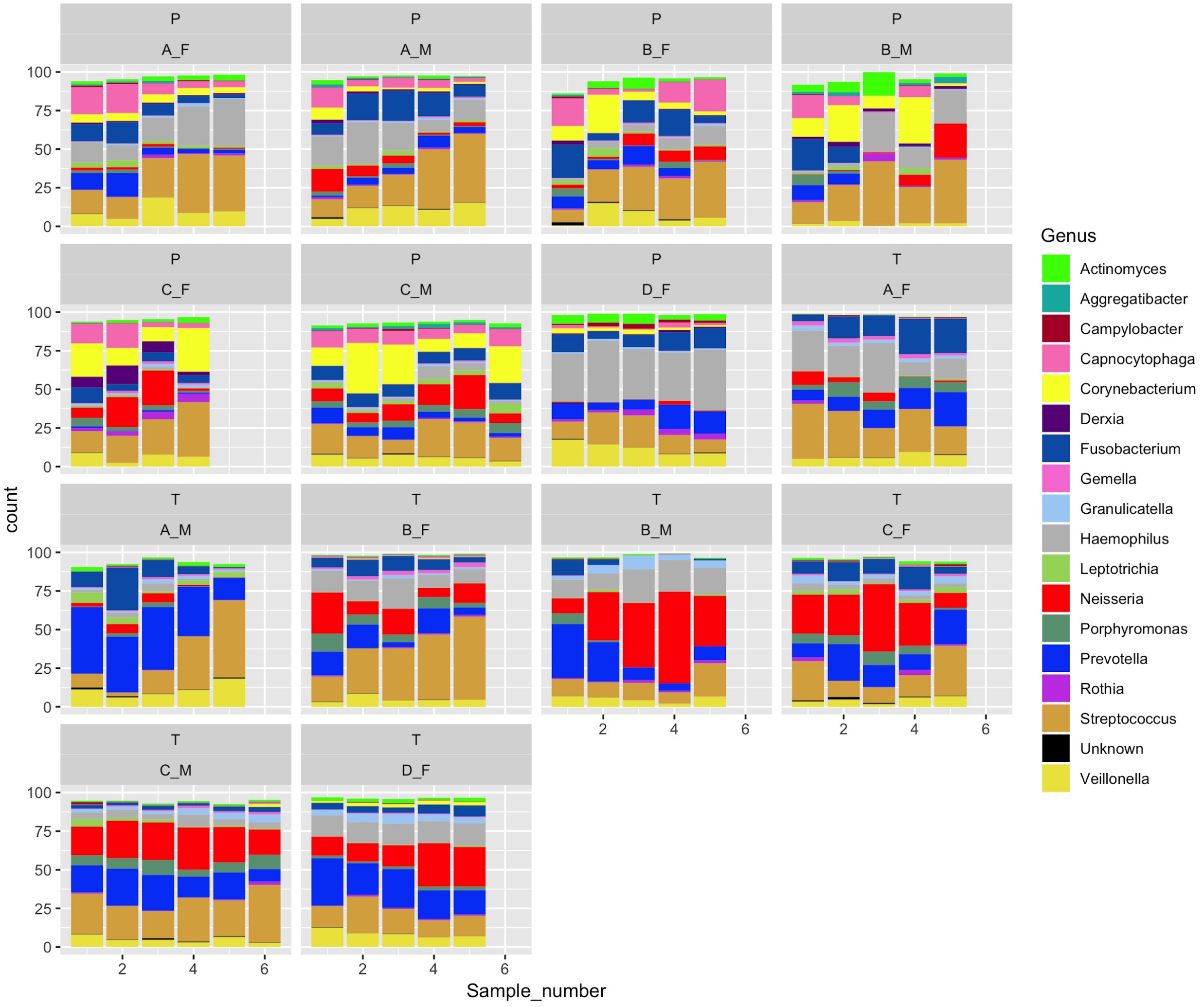

Figure SI1: Taxonomic profiles using 16S rRNA gene amplicon sequence variants (ASVs) produced by MED with taxonomic assignment from GAST.

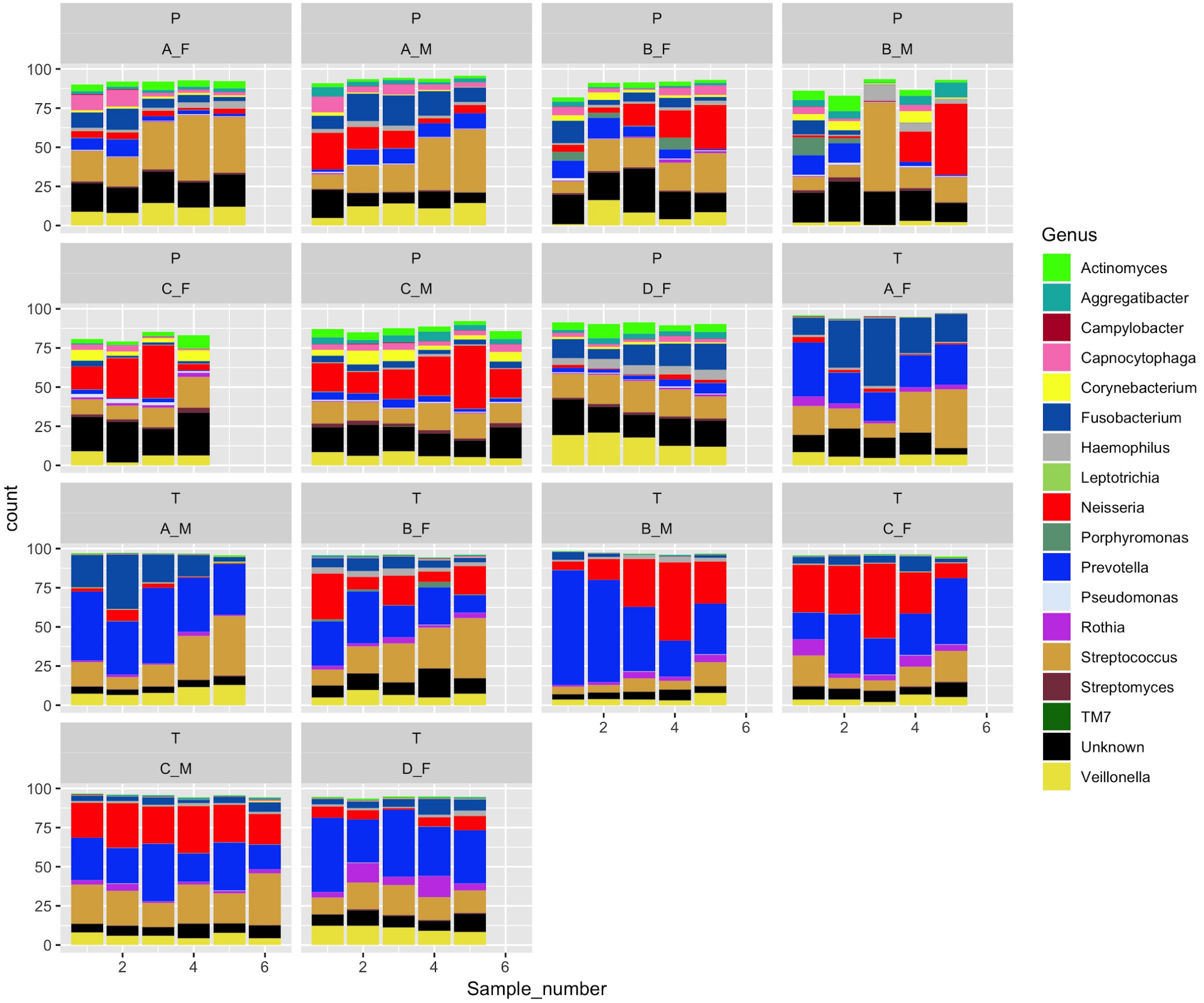

Figure SI2: Taxonomic profiles based on metagenomic short reads using KrakenUniq.

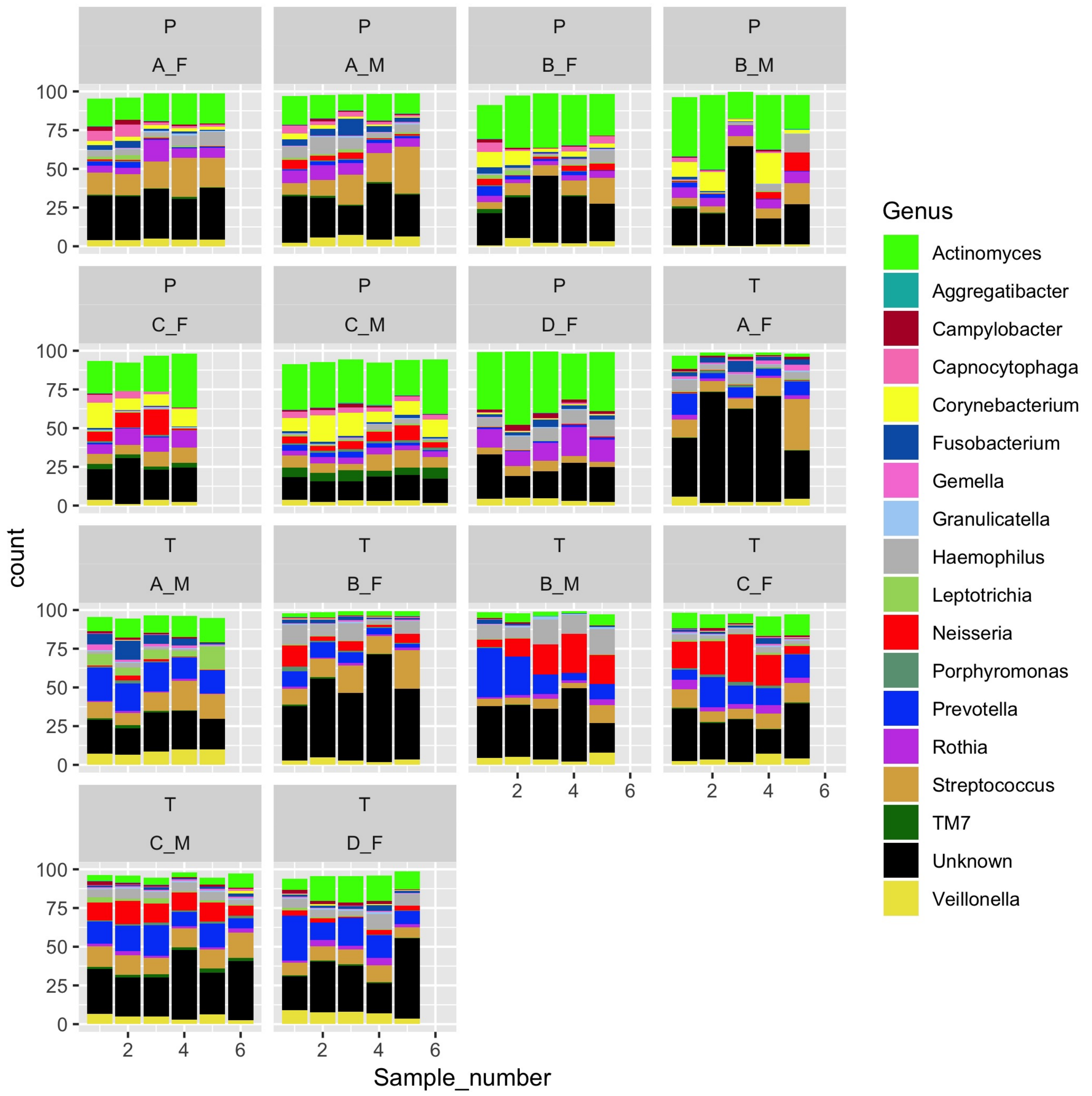

Figure SI3: Taxonomic profiles based on coverages of MAGs.

We note that the amplicon sequences were prepared from the same samples used for shotgun metagenomic sequencing, except that for 3 donors whose amplicons were prepared from tongue swab samples taken in parallel with the scrape samples used for metagenomics. To generate the bar plots per sample and method, we used ggplot2 (Wickham 2016) using Supplementary Tables 2f, 4f, and 5e, which give access to relative abundance data for MAGs, KrakenUniq, and 16S rRNA, respectively.

(B) Average Nucleotide Identity (ANI) of oral TM7

Each of the monophyletic clades that we identified include diverse sub-clades as evident by multiple sub clusters within each clade (see Figure 3 in the main text), hence we sought to search for genomic identity boundaries that could allow the definition of distinct species within these clades. To examine whether phylogenetic clusters within the clades we identified correspond to species of TM7, we computed the average nucleotide identity (ANI) between each pair of genomes. Multiple studies have suggested a 95% cutoff using ANI to determine bacterial species (Jain et al. 2018; Konstantinidis and Tiedje 2005). Our analysis revealed 12 sub-clades that included at least 2 genomes each and separated according to a within-group alignment coverage of >25% and identity >90% (Figure 3, Supplementary Tables 7f, 7g, 7h, and 7i). We hypothesize that each of these represent a separate species, despite the slightly lower than the aforementioned 95% identity cutoff. Genomes of sub-clades T2_a and T2_b aligned between each other with alignment coverage of 50%-70% and identity of 85%-88%, suggesting that these two represent two species of the same genus (Figure 3, Supplementary Table 7h). There were only two other cases in which outgroup members had alignment coverage above 25%. ORAL_P_C_M_Bin_00016 had 30% alignment coverage and 83% identity to ORAL_P_B_M_MAG_00013 (P1_a), suggesting that it could belong to the same genus as the genomes of sub-clade P1_a. Similarly, ORAL_P_C_M_Bin_00022 appears to be a single representative amongst our genomes of a species that belongs to the same genus as P2_b, as it aligned with ~50% coverage and ~85% identity with all four members of P2_b (including TM7x). Since we found no other significant alignment between members of distinct sub-clades, these TM7 genomes potentially represent at least 11 distinct genera.

(C) Occurrence of TM7 across additional oral sample types, other than supragingival plaque and tongue dorsum, and including samples from patients with periodontitis

To examine the occurrence of the TM7 populations across the oral cavity, we used 68 HMP samples with a total of 7 additional sample types (Supplementary Table 7j), as well

as 24 subgingival samples from 9 patients with periodontitis. The number of reads per sample was comparable across sample types with the exception of saliva samples, which had a lower number of reads per sample by an order of magnitude as compared to other sample types (Figure SI4). TM7 populations were detected in all sample types except for the single hard palate sample (Figure SI5, Supplementary Table 4o). While presence of populations in the subgingival plaque mostly matched with their presence in supragingival plaque, some populations were found in a larger portion of the 10 subgingival plaque samples as compared to supragingival plaque (Figure SI5). Moreover, we found that occurrence in subgingival plaque did not imply occurrence in supragingival plaque. For example, from the 5 individuals for which ORAL_P_C_M_Bin_00016 (clade P1) was detected in the subgingival plaque, we only detected this population in the supragingival plaque of one individual. ORAL_P_C_M_MAG_00010 (sub-clade P4_a) also appeared to be enriched in subgingival plaque vs. supragingival plaque. This genome belongs to group 'G5', which has been previously suggested to be enriched in patients with periodontitis based on studies of 16S rRNA amplicons (Abusleme et al. 2013). Our analysis of subgingival samples from patients with periodontitis revealed a similar occurrence as compared to the 10 subgingival plaque samples of the 8 healthy HMP individuals (Figures SI7,SI8 Supplementary Table 7p-s). In palatine tonsils and throat samples we detected only tongue-associated TM7, while in keratinized gingiva samples only members of clade T2 and sub-clade P1_c were detected. ORAL_T_C_M_Bin_00011 (sub-clade T2_c) appeared more prevalent and abundant in keratinized gingiva samples than in tongue samples, and ORAL_T_B_F_Bin_00010 (clade T2) was more abundant in buccal mucosa samples than in tongue samples (Figure SI6, Supplementary Table 4o). Due to the low number of HMP samples per sample type (other than tongue dorsum and supragingival plaque) further investigation would be required in order to confidently determine whether such associations exist.

The paired-end reads of the 24 subgingival plaque samples from patients with periodontitis from the study by Califf et al. (Califf et al. 2017) were received directly from the authors, since the samples that were deposited on MG-RAST with the original Califf et al. publication included only one of the pairs of reads. Raw sequences were analyzed

and the occurrence of TM7 MAGs in these samples were assessed as described in the Methods section of the main text.

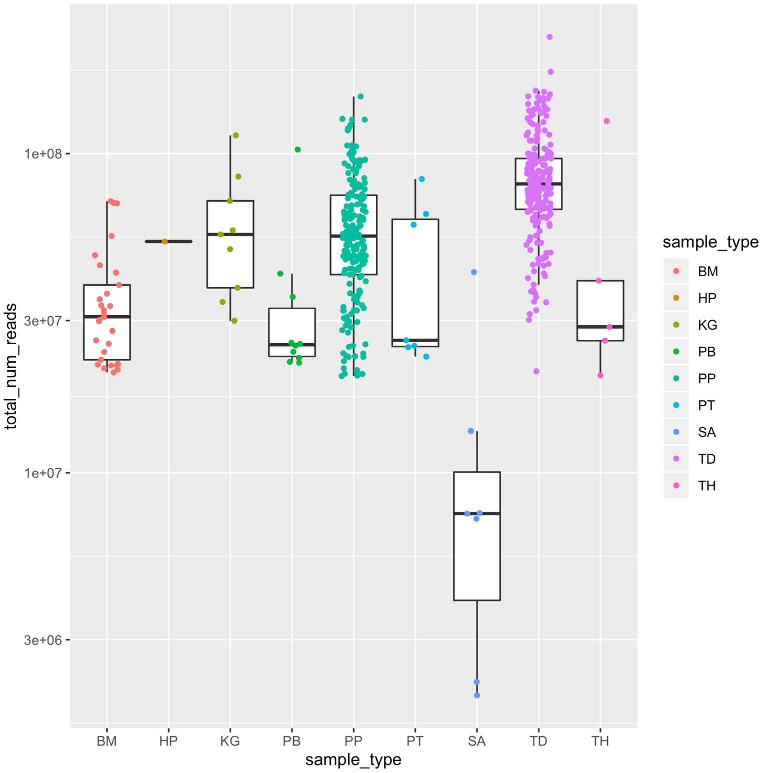

Figure SI4 - number of reads per metagenome. Each data point represents the number of reads in a single sample for the 9 sample types.

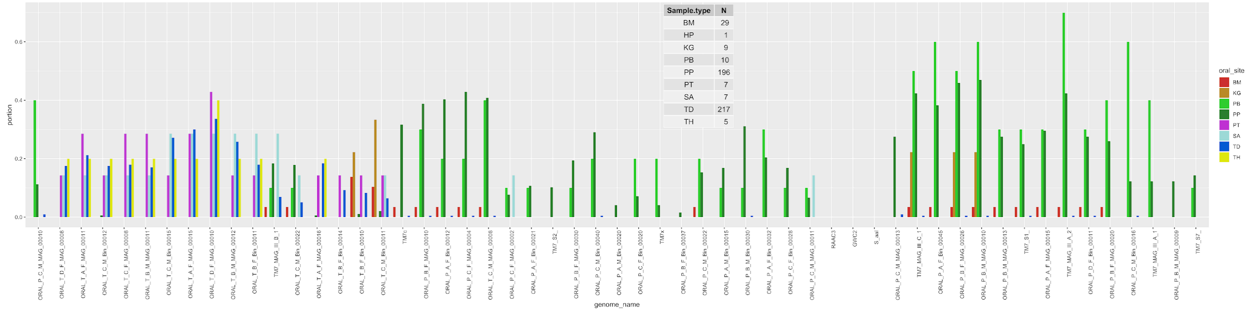

Figure SI5 - Occurrence of TM7 across oral sample types. For each of the 55 genomes (on the x-axis) the colored bars represent the portion of samples per sample type in which it is detected (detection > 0.5).

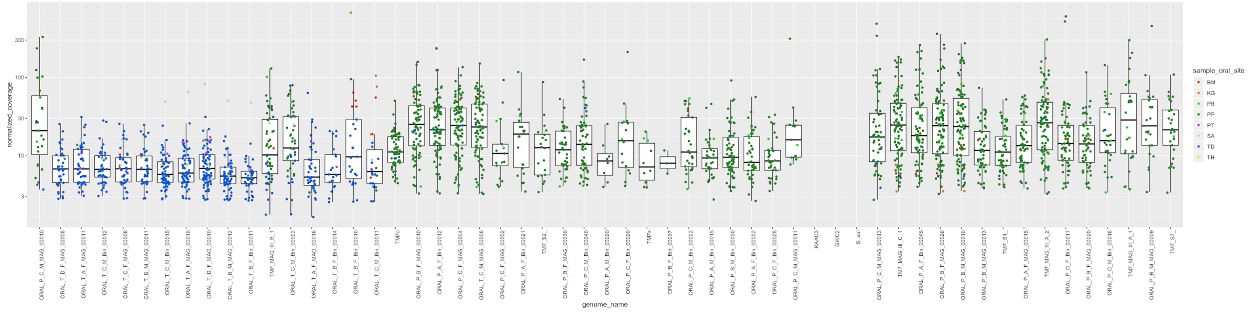

Figure SI6 - Coverage of TM7 across oral sample types. Boxplots of the normalized coverages of each TM7 across samples. Data points are colored according to sample type.

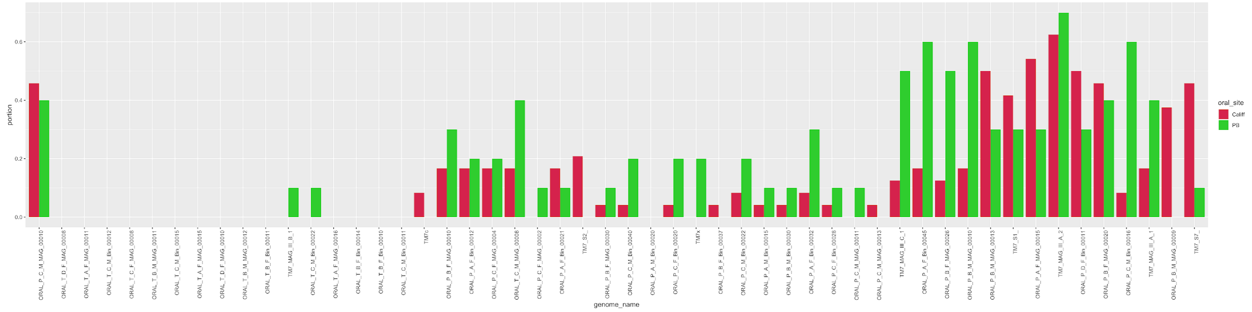

Figure SI7 - Occurrence of TM7 in subgingival plaque samples of healthy individuals and individuals with periodontitis is mostly matching. Bars indicate the portions of subgingival plaque samples from healthy individuals (green) and individuals with periodontitis in which each of the 55 TM7 are detected.

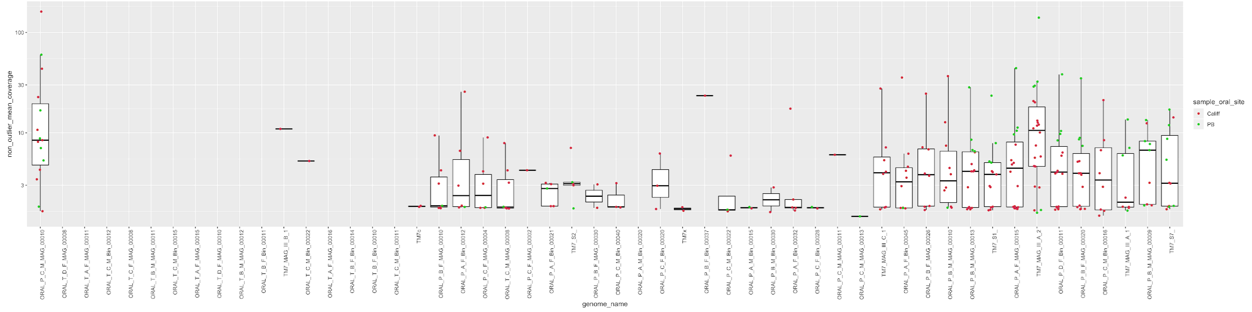

Figure SI8 - Coverage of TM7 in subgingival plaque. Boxplots of the normalized mean coverage of TM7 in samples of healthy individuals (green) and individuals with periodontitis (red).

(D) Mobile elements and prophages in TM7 genomes

In order to systematically search TM7 genomes for evidence of prophages we used VirSorter (Roux et al. 2015) and the “inovirus detector” (Roux et al. 2019) to automatically detect contigs that potentially include prophages in the TM7 genomes and detected 47

contigs with potential prophages (Supplementary Table 8g). We extended this list to a total of 58 contigs by manually identifying additional contigs using functional annotations as markers for phages, and by searching for contigs with GCs that associate with the contigs detected by VirSorter/inovirus detector (Supplementary Table 8g). We manually examined these contigs and identified 36 contigs that include partial or complete prophages, which we manually curated to determine the likely start and end nucleotide positions of the prophages (Supplementary Table 8g). In order to search for conserved sequences amongst these phages, we employed a pangenomic approach. Our pangenomic analysis revealed contigs that likely represent different fragments of the same prophage (Figure SI10); we merged these contigs and removed 9 contigs that were mostly composed of singleton gene clusters to generate a second pangenomic analysis with a refined collection of 25 prophages (Figure SI9). Clustering this refined collection of prophages according to the occurrence of gene clusters revealed 9 “phage groups” of closely related prophages present in two or more TM7 genomes (Figure SI9).

Functional annotation is lacking for most virus genes, and the sequence diversity amongst the viral proteins is high, as is demonstrated in the lack of shared GCs across phages in Figure SI9. Hence, it is challenging to find suitable targets for phylogenetic analysis of phages. In an effort to study the phylogenetic relationships of the phages we used two hallmark genes of (pro)phages: (1) integrase and (2) terminase to compute phylogenies. We performed a phylogenetic analysis using the 13 integrases we identified in our collection of prophages (Figure SI11). Our results reveal cases in which phages that associate with highly divergent hosts rely on similar integrases, while phages that otherwise appear to be closely related (i.e. belong to the same “phage group”) often rely on divergent integrases (Figure SI11). The phylogenetic tree we computed using the 10 tail terminase large subunit identified in the prophages showed a better overall concordance with the organization according to GCs (Figures SI9, SI12). Genomes of phage groups “pg02”, “pg07”, and “pg08” had high within-group identity of the terminase large subunit, but “pg01”, which also shows large variability in the pagenomic analysis (Figure SI9) included prophages with divergent terminase large subunit, despite the fact that their hosts belonged to the same species (P1_a). While it appears that distantly related phages, infecting distantly related hosts, can use very similar integrases (Figure

SI11), our data does not include an case in which distantly related phages harbor similar terminases (Figure SI12). To examine the novelty of these prophages we searched for similar nucleotide sequences using Blast against the NCBI's nr nucleotide collection, but this search had no results, emphasizing the novelty of these sequences.

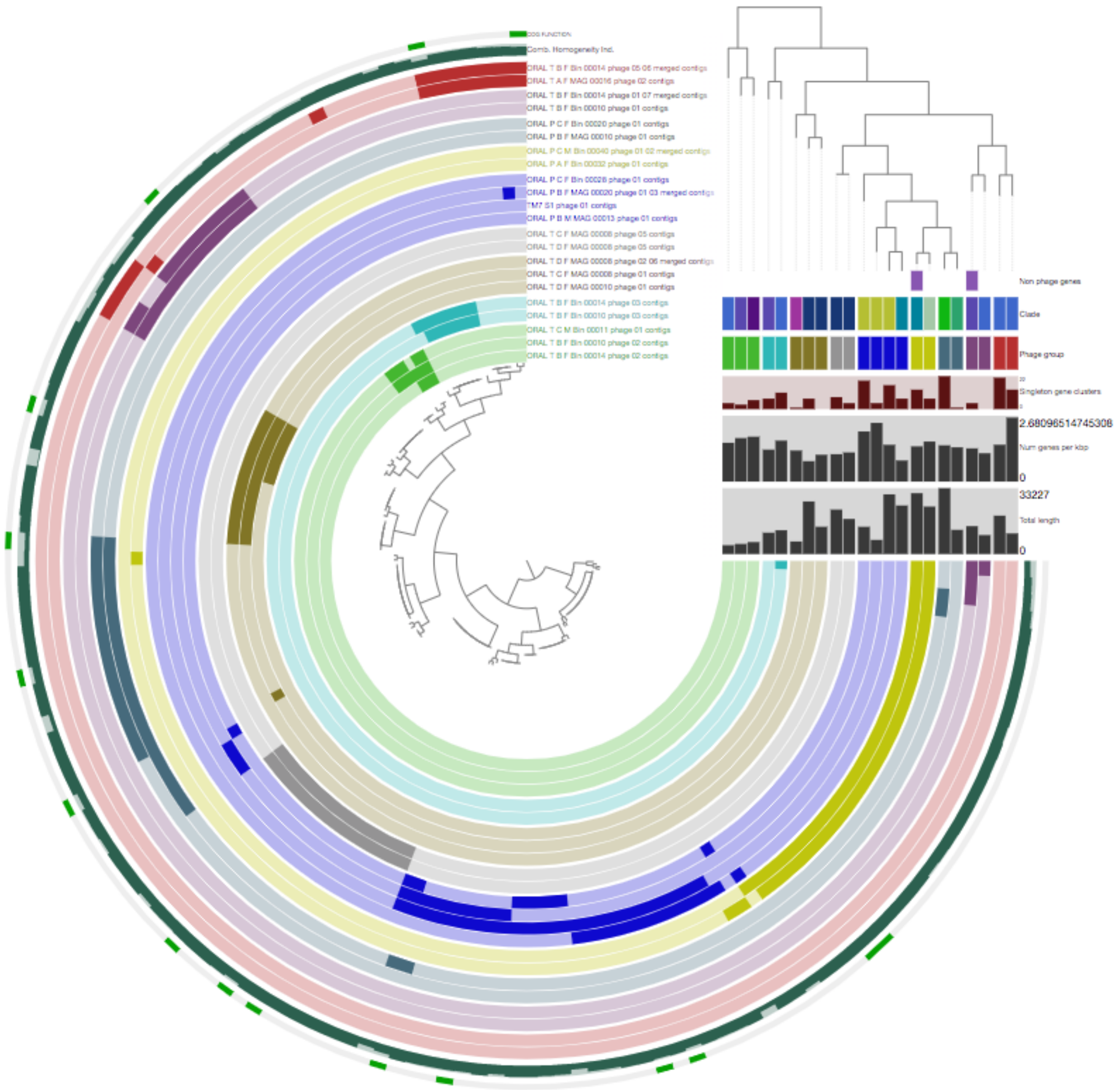

Figure SI9 - Pangenomic analysis of TM7 prophages reveals 9 "phage groups" of closely related phages. The dendrogram at the center of the figure represents the hierarchical clustering, using Euclidean distance and Ward's method, based on the frequency of occurrence of 143 GCs, each containing at least two homologous genes from at least two prophage sequences. The 22 inner circular layers represent prophage sequences, where each data point marks the presence or absence of a protein that belongs to the corresponding GC. Colors of these 22 layers are

according to their “phage group” affiliation. The two outermost circular layers represent the combined homogeneity index for each GC, and the GCs that were annotated with a COG function (green). A low homogeneity index signifies higher sequence diversity amongst the proteins that comprise a GC. The dendrogram at the top right represents the hierarchical clustering of the prophage sequences according to the GC frequency of occurrence using Euclidean distance and Ward’s method. The first horizontal layer below the dendrogram marks the two prophages that include a TM7 protein annotated as “Stress-induced bacterial acidophilic repeat motif”, a core protein of TM7 genomes. The next two layers show the clade affiliation of the TM7 genomes, and the “phage group” affiliation. The lowest three horizontal layers show the number of singletons, number of genes per kbp, and the total length for each prophage sequence.

The recovery of multiple closely related phages from TM7 genomes, as well as the presence of host (TM7) genes on the same contigs that contain the phage genes provide strong evidence for the association of these phages with the TM7 genomes. To further enforce this association, we used CRISPRCasFinder (Couvin et al. 2018) to search the TM7 genomes for CRISPR spacers and survey existing spacers for ones that match our collection of prophages. CRISPRCasFinder identified 66 CRISPR arrays, of which 14 had evidence level 3 or 4 as defined by Couvin et al. (2018) (Supplementary Table 8l), and originated from 12 genomes spanning clades P1, P2, P3, P4, and T2, but not T1 nor any of the environmental genomes. We blasted the set of 14 CRISPR arrays against the TM7 genomes and found a total of 9 spacers with blast hits that were not self-hits (i.e. not a blast match of the spacer to itself), which included 7 spacers with a single external match (i.e. a match outside of the genome where the spacer was found), 1 spacer with two external matches, and 1 spacer with 2 external matches and one internal match, showing that this spacer was self-targeting (Supplementary Table 8m). Five of these 9 spacers had hits to pg01 prophages and revealed that this family of prophages targets a wide variety of TM7 species within the ‘G1’ oral clades P1, P2, and P3 (Supplementary Table 8m). Another spacer matched a pg06 prophage. While we found pg06 prophages in genomes of sub-clades P2_a and P2_c, this spacer was found in a P3_a genome. An additional spacer from a P3_a genome matched a prophage from a P1_a genome suggesting the existence of multiple phage groups that target a variety of ‘G1’ oral genomes. Two additional spacers had hits across G1 genomes, but these matched sequences that we did not identify as prophages and were composed of singleton GCs with no functional annotation, rendering it hard to determine whether these are prophages or other mobile genetic elements. As mentioned above, we found a spacer from P_A_F_Bin_00032 to be self-targeting. Despite being potentially detrimental and

conferring autoimmunity, self-targeting spacers are fairly common (Stern et al. 2010). In this case, the spacer matched 3 of the 4 genes in our dataset that comprise GC_00002421 in P2_a genomes. This GC had no COG function but was recognized to have a 'PEGA domain' by Pfam, which is found in surface layer proteins. While this GC was unique to members of P2_a, it seems that this protein is conserved and represents a core function in the TM7 pangenome, since a protein with this annotation was found in nearly all genomes, and almost always flanked by a "Sortase (surface protein transpeptidase)". The apparent viability of the P_A_F_Bin_00032 population as evident by the recovery of the genome, despite the CRISPR self-targeting of a core function might suggest that this core function is not strictly required for the survival of TM7 in the oral cavity.

In contrast to the oral clades P1, P2, P3, P4, and T2, we found no evidence for CRISPR-cas systems in T1 genomes nor in the three environmental genomes. The CRISPRCasFinder output included contigs from T1 genomes, but these only had evidence level 1 or 2, suggesting that they could be spurious identifications (Supplementary Table 8l). Indeed, many of these appeared to fall within genes that belong to a single GC, suggesting that something about the sequence of these specific genes confuses the CRISPRCasFinder algorithm. There was only one contig from one of the three environmental genomes (GWC2) that was included in the output of CRISPRCasFinder, but it had evidence level 1, and the identification fell within a TM7 core protein, and hence is likely an erroneous identification. In accordance with the lack of CRISPR arrays, we did not find any of the CRISPR associated proteins in the environmental genomes nor in genomes of clade T1, but we did find these proteins in genomes of the oral clades P1, P2, P3, P4, and T2. We find the lack of prophages and the lack of CRISPRs in environmental genomes to be highly interesting, since these fall within the G1 group to which the P1, P2, and P3 clades belong, which could imply that these CRISPR-cas systems are unique to oral-associated (or more generally to animal-associated) TM7, but an analysis of a wider variety of environmental TM7 would be required to test this hypothesis. To search for the potential source for CRISPR proteins in oral TM7, we blasted cas9 proteins from 6 genomes representing all 5 CRISPR-containing clades, and representing the three GCs annotated as cas9 proteins, against

the NCBI's nr protein sequences. All 6 cas9 proteins were matching the same collection of proteins from oral TM7, but no environmental TM7. The top non- TM7 matches were of Firmicutes (Bacilli and Clostridia), suggesting that these proteins were once horizontally transferred from Firmicutes to oral-associated TM7. Future investigations could include a phylogenetic analysis of CRISPR associated proteins of TM7 along with ones from other CPR and non-CPR (including human-associated) genomes to further shed light on the source of CRISPR systems in TM7 genomes, and whether these are unique to mammalian-associated TM7.

While T1 and environmental genomes lacked CRISPR-cas systems, they could alternatively rely on restriction modification systems to defend against phages. Based on COG annotations, we identified Type I and/or Type II restriction-modification systems in 34 TM7 genomes spanning all identified oral clades and two of the three environmental genomes, GWC2 and RAAC3. In addition to lacking CRISPR-cas systems, members of clade T1 were also lacking a protein annotated with the COG function "Phage shock protein PspC (stress-responsive transcriptional regulator)", which was found in nearly all genomes from all other oral clades and in two of the three environmental genomes.

In addition to prophages, we identified other mobile genetic elements in many TM7 genomes. 33 genes coding for various transposases were detected in 18 genomes, covering all oral clades and the three environmental TM7. These genes comprised a total of 22 GCs, and up to four transposases per genome (Supplementary Table 8n). The transposases were predominantly associated with GCs unique to specific lineages. 19 of the 22 GCs were singletons (i.e. identified in a single genome), the three other GCs, GC_00003909, GC_00002371, and GC_00001084 were identified in two, three and seven genomes, respectively. GC_00001084 was annotated as an "ISXO2-like transposase domain" by Pfam and was identified in most P3_a and three P1_b genomes. GC_00002371 was identified in 3 (out of 5) T1_a genomes and was annotated with the COG function "Transposase InsO and inactivated derivatives". While the transposases in T1_a genomes were highly conserved in protein sequences, they occurred in differing positions within the genomes (Supplementary Table 8a), suggesting recent mobility of these elements. GC_00003909 was detected in the two P1_c genomes with the COG

function “Transposase and inactivated derivatives, IS30 family”. In both P1_c genomes, this transposase occurred in the same exact position within the genome, suggesting that this might represent an inactive transposon.

In order to examine the potential origin of the TM7 transposases, we searched for similar sequences in NCBI’s non-redundant protein sequence database (Supplementary Table 8o). The vast majority matched best to transposases from other TM7 genomes or other CPR genomes, including many genomes recovered from environmental samples. For example, the single transposase from T_C_M_MAG_00008 had best matches to other oral TM7, but also matched many other CPR, including CPR MAGs recovered by Probst et al. from an aquifer (Probst et al. 2018). In contrast, T_C_M_Bin_00011 included what appears to be only the N-terminal region of an IS30-family transposase which matched best to transposases from a *Streptococcus agalactiae* genome (89% coverage and 52% identity in protein sequence). Examination of the contig on which this transposase was detected showed that it is not likely to be explained by a binning error, as this transposase was flanked by many core proteins of TM7 on one side, but on the other side, it was flanked by three short proteins that belonged to singleton GCs (i.e. with no homologs in the TM7 pangenome) and no functional annotation (gene IDs 21837-21839 in Supplementary Table 8a). A blast search of protein sequences matched these three proteins with a surprisingly high identity (94%-100%) to genes from other oral bacteria representing various phyla, including Firmicutes, Fusobacteria, and Proteobacteria. The presence of a partial transposase next to genetic elements that appear to be widely shared between oral microbes could reflect a mechanism for horizontal gene transfer between TM7 and non-CPR oral microbes but requires further validation. In summary, these results suggest that the transposases carried by oral TM7 genomes are predominantly anciently associated with CPR genomes, but also include transposases that were likely transferred to oral TM7 from other mammalian-associated bacteria more recently, and could potentially be used to incorporate proteins that are widely shared by oral bacteria.

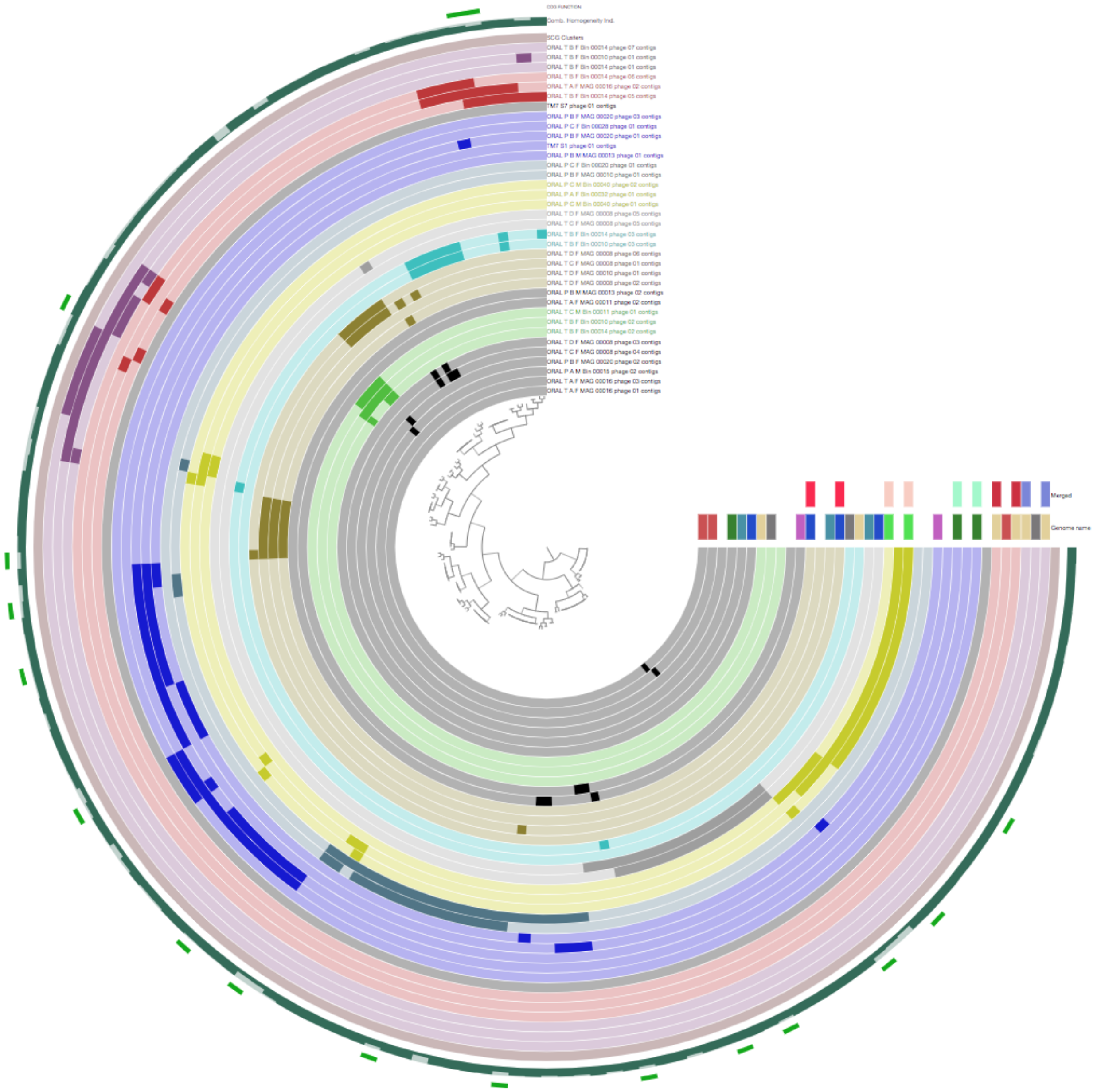

Figure SI10 - Pangenomic analysis of potential prophages includes multiple contigs that likely represent fragments of the same prophage. The gene content of each prophage is represented by an individual layer, and the 9 main groups of TM7-associated prophages are highlighted in different colors across layers. Layers that are in black color are ones that consisted mostly of singletons and were hence excluded from subsequent analysis. On the top right of the figure, the color bars in the top horizontal layer highlight pairs of contigs that belong to the same genome and that we identified as fragments of the same prophage and merged for the subsequent pangenomic analysis (Figure 5). The next horizontal layer identifies prophage sequences that are associated with the same genome.

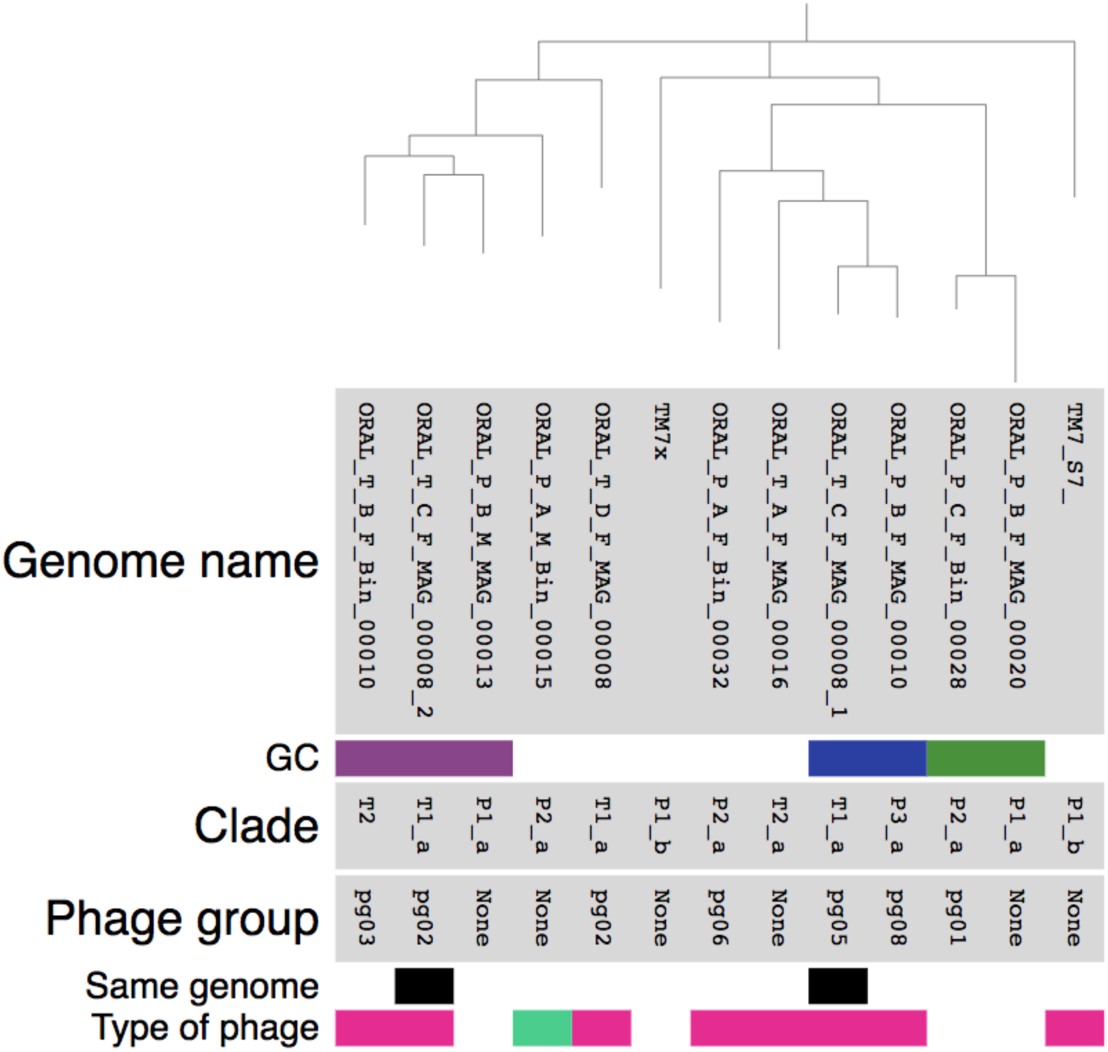

Figure SI11 - phylogeny of phages based on integrases. The dendrogram at the top of the figure represents the maximum likelihood phylogenetic tree of the prophages based on protein sequences of integrases. The names of genomes in which the phage was identified appear below the dendrogram, and a suffix of "_1" and "_2" marks the two prophages that were identified in T_C_F_MAG_00008. "GC": marks the integrases that were in non-singleton GCs. "Clade": the clade or subclade (if one exists) association of the host of each prophage. "Phage group": phage group designation. "Same genome": highlights two prophages from T_C_F_MAG_00008. "Type of phage": either inovirus (green) or caudovirales (pink).

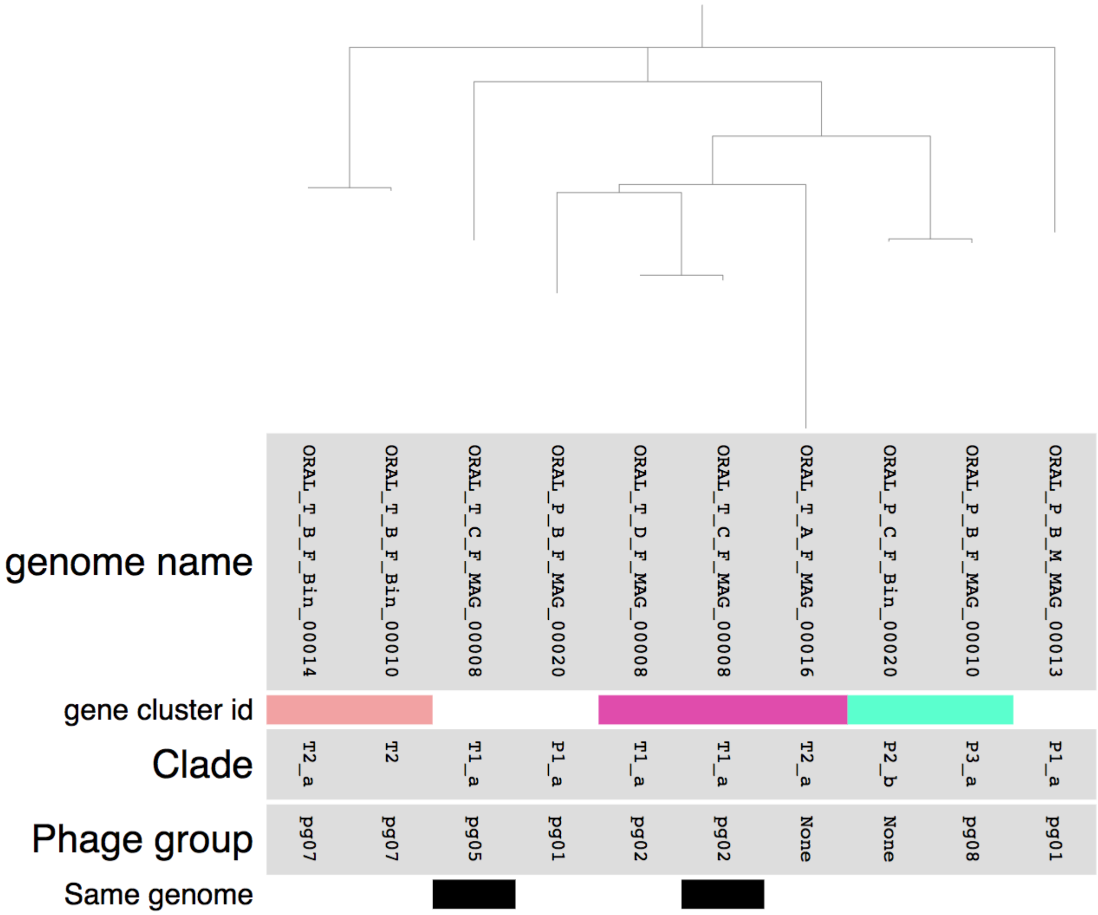

Figure SI12 - phylogeny of phages based on terminases. The dendrogram at the top of the figure represents the maximum likelihood phylogenetic tree of the prophages based on protein sequences of terminase large subunit. The names of genomes in which the phage was identified appear below the dendrogram. “Gene cluster id”: marks the integrases that were in non-singleton GCs. “Clade”: the clade or subclade (if one exists) association of the host of each prophage. “Phage group”: phage group designation. “Same genome”: highlights two prophages from T_C_F_MAG_00008.

(E) Novel non-CPR MAGs

Our collection of MAGs included 43 genomes with no closely related genome in HOMD (Figure 1, Supplementary Table 10a). In order to test the novelty of these genomes, we blasted the protein sequences of the ribosomal proteins of these populations against the NCBI non redundant protein sequences database. In conjunction with the phylogenetic analysis (Figure 1), blast results confirmed that 34 of these genomes represent 11 lineages with no representation on NCBI (from here on referred to as “novel MAGs”), while the additional 9 genomes belong to two lineages from the family Eubacteriaceae

and matched genomes of *Stomatobaculum longum* and *Lachnospiraceae* bacterium oral taxon 096 in NCBI, which were absent from the HOMD at the time that we downloaded the HOMD genomes, but have since been added (Supplementary Tables 10b, 10c).

(F) A novel MAG for a member of the Mollicutes

Members of the Mollicutes, a class of bacteria that lack a cell wall (Davis et al. 2013) are known to be commonly found in the human oral cavity. In particular, Mycoplasma are ubiquitous members of the oral microbiome (Dewhirst et al. 2010) and include some pathogens. Studies based on 16S rRNA amplicons identified two taxa, HMT-504 and HMT-906, as potential members of the Mollicutes on a deep phylogenetic branch between other known Mollicutes and members of the class Erysipelotrichia (Dewhirst et al. 2010). T_C_F_MAG_00011 has no closely related genome on GenBank (Supplementary Table 10c) and our phylogenomic analysis with representatives of all taxa under the classes Mollictutes and Erysipelotrichia as available on GenBank on 12/24/2018. (Figure SI13) placing it deeply branching between these two classes, suggesting it could represent either HMT-504 or HMT-906. Notice that we excluded two GenBank genomes annotated as Erysipelotrichia (GCF.900120365.1, GCF.000178255.1) from our analysis, since our preliminary phylogenetic analysis showed these are likely not members of Erysipelotrichia. The closest genomes to T_C_F_MAG_00011 were members of the genus *Acholeplasma*, including many plant pathogens, but also including a horse oral pathogen (Atobe, Watabe, and Ogata 1983). Our analysis using the HMP metagenomes showed that T_C_F_MAG_00011 is associated with the tongue and occurs in 20% of HMP individuals for which tongue samples are available (Figure S7a, Supplementary Table 10c).

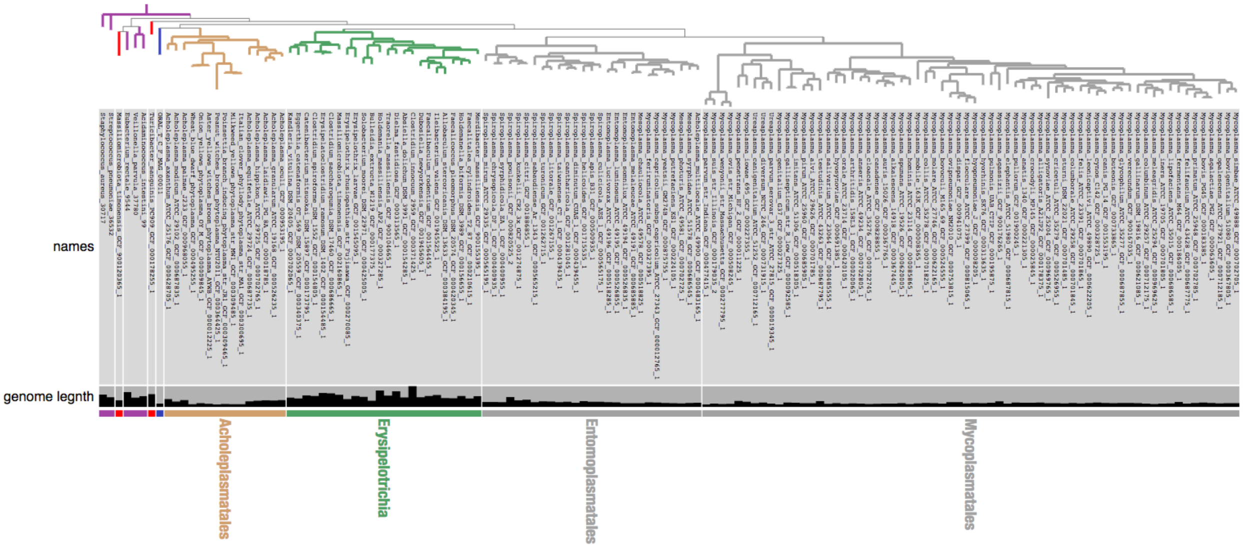

Figure SI13 - phylogeny based on ribosomal proteins places T_C_F_MAG_00011 closest to genomes of Acholeplasmatales. Phylogenetic tree of T_C_F_MAG_00011 (blue) together with RefSeq genomes of class Erysipelotrichia (green), phylum Tenericutes, including class Mollicutes, and within it orders Entomoplasmatales and Mycoplasmatales (grey), and Acholeplasmatales (brown), along with five other Firmicutes, representing classes Bacilli, Clostridiales, and Negativicutes as outliers to root the phylogeny (purple). Two genomes wrongly annotated as Erysipelotrichia appear in red color.

(G) Novel Clostridiales MAGs represent prevalent tongue-associated populations

We also have recovered five Clostridiales MAGs for which we could not assign a family designation (Figure SI14). Three MAGs were closely related and seem to represent a prevalent tongue-associated species and were detected in >50% of HMP tongue metagenomes (Figure S7a, Supplementary Tables 10e-h). In addition, we detected T_A_M_MAG_00009 in 30% of tongue samples and 20% of plaque samples, while T_C_M_MAG_00006 was detected only in seven HMP tongue samples (3%), and were each distant phylogenetically from any other genome on our phylogenomic analysis using all Clostridiales genomes available from RefSeq on 9/25/2019.

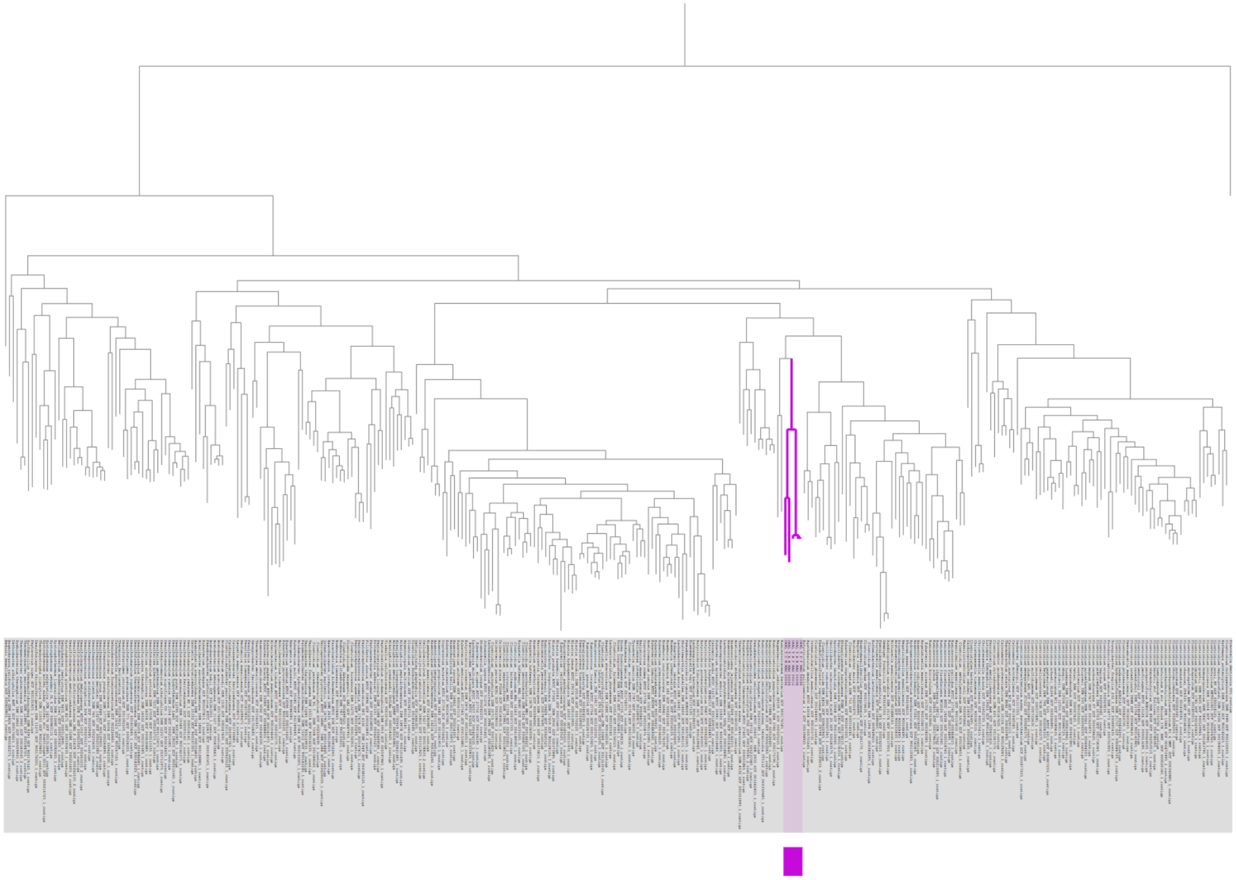

Figure SI14 - phylogenomic analysis of Clostridiales genomes from NCBI with our Clostridiales MAGs. A maximum likelihood phylogenetic tree was computed based on our collection of ribosomal proteins using representative genomes for all taxa of order Clostridiales in RefSeq. Our MAGs are highlighted with purple color. The tree was rooted using a *Prevotella* genome.

(H) Novel Bacteroidia MAGs include a tongue-specialist and a subgingival plaque specialist

One of our Bacteroidia MAGs (P-A-M_MAG_00010) matched a genome recently recovered from a metagenomic sample of periodontal pockets of a patient with periodontitis (McLean et al. 2015) and seems to represent the same species. McLean et al. named this population *Candidatus Bacteroides periocalifornicus* (CBP), although phylogenomic analyses show that it is not a member of the genus *Bacteroides* (McLean et al. 2015). Torres et al. (Torres et al. 2019) showed that this CBP is enriched in subgingival plaque samples as compared to supragingival plaque samples, which our analysis also confirms (Figure S7b-c), an expected result as both analyses relied on the

same HMP samples. Two closely related Bacteroidia (T_B_M_MAG_00007, T_C_F_MAG_00010) were prevalent in our tongue samples and detected in 40% of HMP tongue samples (Figure S7a, Supplementary Table 10f). CBP was the closest relative to these MAGs, but with an average of 76% identity in amino-acid sequences of ribosomal proteins, suggesting that these two lineages are distant and potentially represent distinct genera or families within Bacteroidia.

References

Abusleme, Loreto, Amanda K. Dupuy, Nicolás Dutzan, Nora Silva, Joseph A. Burleson, Linda D. Strausbaugh, Jorge Gamonal, and Patricia I. Diaz. 2013. “The Subgingival Microbiome in Health and Periodontitis and Its Relationship with Community Biomass and Inflammation.” *The ISME Journal* 7 (5): 1016–25.

Atobe, Hisae, Junko Watabe, and Manabu Ogata. 1983. “Acholeplasma Parvum, a New Species from Horses.” *International Journal of Systematic and Evolutionary Microbiology* 33 (2): 344–49.

Breitwieser, F. P., D. N. Baker, and S. L. Salzberg. 2018. “KrakenUniq: Confident and Fast Metagenomics Classification Using Unique K-Mer Counts.” *Genome Biology* 19 (1): 198.

Califf, Katy J., Karen Schwarzberg-Lipson, Neha Garg, Sean M. Gibbons, J. Gregory Caporaso, Jørgen Slots, Chloe Cohen, Pieter C. Dorrestein, and Scott T. Kelley. 2017. “Multi-Omics Analysis of Periodontal Pocket Microbial Communities Pre- and Posttreatment.” *mSystems*. https://doi.org/10.1128/msystems.00016-17.

Couvin, David, Aude Bernheim, Claire Toffano-Nioche, Marie Touchon, Juraj Michalik, Bertrand Néron, Eduardo P. C. Rocha, Gilles Vergnaud, Daniel Gautheret, and Christine Pourcel. 2018. “CRISPRCasFinder, an Update of CRISRFinder, Includes a Portable Version, Enhanced Performance and Integrates Search for Cas Proteins.” *Nucleic Acids Research* 46 (W1): W246–51.

Davis, James J., Fangfang Xia, Ross A. Overbeek, and Gary J. Olsen. 2013. “Genomes of the Class Erysipelotrichia Clarify the Firmicute Origin of the Class Mollicutes.” *International Journal of Systematic and Evolutionary Microbiology* 63 (Pt 7): 2727–41.

Dewhirst, Floyd E., Tuste Chen, Jacques Izard, Bruce J. Paster, Anne C. R. Tanner, Wen-Han Yu, Abirami Lakshmanan, and William G. Wade. 2010. “The Human Oral Microbiome.” *Journal of Bacteriology* 192 (19): 5002–17.

Eloe-Fadrosh, Emiley A., Natalia N. Ivanova, Tanja Woyke, and Nikos C. Kyrpides. 2016. “Metagenomics Uncovers Gaps in Amplicon-Based Detection of Microbial Diversity.” *Nature Microbiology*. https://doi.org/10.1038/nmicrobiol.2015.32.

Eren, A. Murat, Lois Maignien, Woo Jun Sul, Leslie G. Murphy, Sharon L. Grim, Hilary G. Morrison, and Mitchell L. Sogin. 2013. Oligotyping: Differentiating Between Closely Related Microbial Taxa Using 16S rRNA Gene Data. *Methods in Ecology and Evolution* 4(12):1111-1119.

Eren, A. Murat, Hilary G. Morrison, Pamela J. Lescault, Julie Reveillaud, Joseph H. Vineis, and Mitchell L. Sogin. 2015. “Minimum Entropy Decomposition: Unsupervised Oligotyping for Sensitive Partitioning of High-Throughput Marker Gene Sequences.” *The ISME Journal* 9 (4): 968–79.

Escobar-Zepeda, Alejandra, Elizabeth Ernestina Godoy-Lozano, Luciana Raggi, Lorenzo Segovia, Enrique Merino, Rosa María Gutiérrez-Rios, Katy Juarez, Alexei F. Licea-Navarro, Liliana Pardo-Lopez, and Alejandro Sanchez-Flores. 2018. “Analysis of Sequencing Strategies and Tools for Taxonomic Annotation: Defining Standards for Progressive Metagenomics.” *Scientific Reports* 8 (1): 12034.

Huse, Susan M., Les Dethlefsen, Julie A. Huber, David Mark Welch, David A. Relman, and Mitchell L. Sogin. 2008. “Exploring Microbial Diversity and Taxonomy Using SSU rRNA Hypervariable Tag Sequencing.” *PLoS Genetics* 4 (11): e1000255.

Jain, Chirag, Luis M. Rodriguez-R, Adam M. Phillippy, Konstantinos T. Konstantinidis, and Srinivas Aluru. 2018. “High Throughput ANI Analysis of 90K Prokaryotic Genomes Reveals Clear Species Boundaries.” *Nature Communications*. https://doi.org/10.1038/s41467-018-07641-9.

Konstantinidis, Konstantinos T., and James M. Tiedje. 2005. “Genomic Insights That Advance the Species Definition for Prokaryotes.” *Proceedings of the National Academy of Sciences of the United States of America* 102 (7): 2567–72.

McLean, Jeffrey S., Quanhui Liu, John Thompson, Anna Edlund, and Scott Kelley. 2015. “Draft Genome Sequence of ‘ Candidatus Bacteroides Periocalifornicus,’ a New Member of the Bacteriodetes Phylum Found within the Oral Microbiome of Periodontitis Patients.” *Genome Announcements*. https://doi.org/10.1128/genomea.01485-15.

Probst, Alexander J., Bethany Ladd, Jessica K. Jarett, David E. Geller-McGrath, Christian M. K. Sieber, Joanne B. Emerson, Karthik Anantharaman, et al. 2018. “Differential Depth Distribution of Microbial Function and Putative Symbionts through Sediment-Hosted Aquifers in the Deep Terrestrial Subsurface.” *Nature Microbiology* 3 (3): 328–36.

Roux, Simon, Francois Enault, Bonnie L. Hurwitz, and Matthew B. Sullivan. 2015. “VirSorter: Mining Viral Signal from Microbial Genomic Data.” *PeerJ* 3 (May): e985.

Roux, Simon, Mart Krupovic, Rebecca A. Daly, Adair L. Borges, Stephen Nayfach, Frederik Schulz, Allison Sharrar, et al. 2019. “Cryptic Inoviruses Revealed as Pervasive in Bacteria and Archaea across Earth’s Biomes.” *Nature Microbiology*, July. https://doi.org/10.1038/s41564-019-0510-x.

Stern, Adi, Leeat Keren, Omri Wurtzel, Gil Amitai, and Rotem Sorek. 2010. “Self-Targeting by CRISPR: Gene Regulation or Autoimmunity?” *Trends in Genetics: TIG* 26 (8): 335–40.

Torres, Pedro J., John Thompson, Jeffrey S. McLean, Scott T. Kelley, and Anna Edlund. 2019. “Discovery of a Novel Periodontal Disease-Associated Bacterium.” *Microbial Ecology* 77 (1): 267–76.

Wickham, Hadley. 2016. *ggplot2: Elegant Graphics for Data Analysis*. Springer.
