# Supplementary material for: Functional and genetic markers of niche partitioning among enigmatic members of the human oral microbiome
Source: Genome Biol. 2020 Dec 16;21:292. doi: 10.1186/s13059-020-02195-w (PMC7739484; doi:10.1186/s13059-020-02195-w)
Supplement: Supplementary file 13 — Additional file 13. Review history. [file 13059_2020_2195_MOESM13_ESM.docx]

Review History

**First round of review**

**Reviewer 1**

**Are you able to assess all statistics in the manuscript, including the appropriateness of statistical tests used?** Yes, and I have assessed the statistics in my report.

**Comments to author:**

This is an extremely well-written manuscript with a careful analysis of metagenomic data derived from two distinct ecological niches in the oral cavity. The authors employ detailed pan- and phylogenetic analysis with a focus on members of the candidate phylum TM7 to reveal distinct clades associated with tongue and supragingival plaque and provide insight into potential different evolutionary origins. The authors explain their analyses in great detail with adequate consideration of limitations. In addition, this study provides a wealth of well-curated novel genomes, which allowed of new taxonomic lineages. The findings, methods and approaches detailed in this manuscript could easily be translated to the exploration of different microbial ecological niches and are therefore of great importance for the field at large.

Minor revisions:

The color schemes (and font size) chosen in some of the figures do not allow easy visual distinction. Especially the "hues of green" chosen for "plaque" vs "plaque and tongue" in Figs 2 - 6. Labeling of the supplemental figures 2a and 2b, 5b, 5c, 6b, 6c, 7b, 7c (and others) should have larger font size for better visibility

**Reviewer 2**

**Are you able to assess all statistics in the manuscript, including the appropriateness of statistical tests used?** Yes, and I have assessed the statistics in my report.

**Comments to author:**

In this study, the authors use metagenomic genome assembly from tongue and plaque samples to gain insight on understudied members of the human oral microbiome. The paper has a focus on Saccharibacteria, a.k.a. TM7, though they discuss other members of the candidate phyla radiation and newly observed species from well-established phyla.

The main strength of the manuscript is the authors' comprehensive analysis of metagenome-assembled genomes, and their attention to using the analysis results to bear on questions of bacterial ecology and evolution. The list analysis methods applied in the paper would serve as a solid template for other researchers studying the human microbiome using assembly-based approaches.

Although the research is well done and the paper is well-organized, the manuscript requires substantial attention to tighten up the writing. The paper suffers from overly complex sentences and paragraphs that lack a clearly stated topic. The main ideas of this manuscript are important and deserve to be communicated clearly and succinctly. In the comments below, I will point out areas that need particular attention, but I want to emphasize that the paper as a whole would benefit from an effort to simplify sentences and focus paragraphs on a clear topic.

Major comments:

Line 219. I notice that the subject ID's consist of two letters, and want to double check with the authors to make absolutely sure that the subject ID's do not contain any identifiable or protected health information about the study participants.

Line 218-231, 281-285. The authors have done well to both point out a general rule (TM7 populations were exclusive to plaque or tongue), and discuss an exception to the rule (except for T_C_M_Bin_00022). In areas where the authors discuss a general rule and highlight an exception, the text needs to be cleaned up so that it is clear when authors switch from one topic to another.

Line 258-341. This subsection seems to lose its way and becomes unfocused. It would benefit substantially from clear topic sentences, shorter paragraphs, and fewer dependent phrases.

Line 404-405. The analyses presented in the manuscript use several types of clustering: clustering of metagenomes, clustering of homologous genes, and clustering of GCs by presence/absence among the metagenomes. The authors should adopt a clear and consistent language to discuss each type of clustering, and signal clearly when the discussion switches from one to another.

Line 599-671. The paper has such a focus on TM7 that I wonder if it shouldn't be mentioned in the title.

Line 681-682. How can other researchers use your important results and new genomes in future work? By carrying out this study, you have added to the data resources available to researchers in this area. Please let us know how we can pick up your data and use it in the next study.

Minor comments:

Line 164. If assemblies from plaque samples recruited more reads overall relative to tongue samples, could this be due to the relative abundance of Streptococcus or other common oral bacteria? I imagine that the presence of several closely related species of Streptococcus could diminish the assembly quality and reduce the total number of reads recruited onto contigs.

Line 180. Is there a danger that your criteria for inclusion of metagenomes introduces a bias favoring small genomes?

Line 193. I appreciate that the authors manually refined the metagenomes to guard against contamination. It would be useful for the reader to know how many contaminated genomes were corrected in this way, and what qualities of the assembly led the authors to make the correction.

Line 252-257. Here you have a group of sentences that clearly summarize the results of the subsection. I suggest that this be its own paragraph, beginning with, "The phylogenomic analysis…"

Line 359-387. This subsection is written as one paragraph, but clearly covers three topics: (1) what you set out to do, (2) what you did for quality control, and (3) what the results were. Your important results are getting lost in the middle of a long paragraph.

Line 493. "nearly all genomes" -- no reason to be vague here. How many?

**Authors Response**

**Point-by-point responses to the reviewers’ comments:**

Reviewer #1

This is an extremely well-written manuscript with a careful analysis of metagenomic data derived from two distinct ecological niches in the oral cavity. The authors employ detailed pan- and phylogenetic analysis with a focus on members of the candidate phylum TM7 to reveal distinct clades associated with tongue and supragingival plaque and provide insight into potential different evolutionary origins. The authors explain their analyses in great detail with adequate consideration of limitations. In addition, this study provides a wealth of well-curated novel genomes, which allowed of new taxonomic lineages. The findings, methods and approaches detailed in this manuscript could easily be translated to the exploration of different microbial ecological niches and are therefore of great importance for the field at large.

***Author Response***

***We thank the reviewer for their careful reading and evaluation of our study.***

Reviewer #1 comment #1

The color schemes (and font size) chosen in some of the figures do not allow easy visual distinction. Especially the "hues of green" chosen for "plaque" vs "plaque and tongue" in Figs 2 - 6.

***Author response***

***We addressed this issue by changing the color of “plaque and tongue” to turquoise in all figures. It now looks significantly more distinguishable.***

Reviewer #1 comment #2

Labeling of the supplemental figures 2a and 2b, 5b, 5c, 6b, 6c, 7b, 7c (and others) should have larger font size for better visibility

***Author response***

***We edited our figures to switch to a larger font size. In addition to the supplemental figures the reviewer mentioned (2a, 2b, 5b, 5c, 6b, 6c, 7b, and 7c), we also updated supplemental figures 5a, 6a, 4c, and 8 to improve readability and clarity.***

Reviewer #2, general comments

In this study, the authors use metagenomic genome assembly from tongue and plaque samples to gain insight on understudied members of the human oral microbiome. The paper has a focus on Saccharibacteria, a.k.a. TM7, though they discuss other members of the candidate phyla radiation and newly observed species from wellestablished phyla.

The main strength of the manuscript is the authors' comprehensive analysis of metagenome-assembled genomes, and their attention to using the analysis results to bear on questions of bacterial ecology and evolution. The list analysis methods applied in the paper would serve as a solid template for other researchers studying the human microbiome using assembly-based approaches.

Although the research is well done and the paper is well-organized, the manuscript requires substantial attention to tighten up the writing. The paper suffers from overly complex sentences and paragraphs that lack a clearly stated topic. The main ideas of this manuscript are important and deserve to be communicated clearly and succinctly. In the comments below, I will point out areas that need particular attention, but I want to emphasize that the paper as a whole would benefit from an effort to simplify sentences and focus paragraphs on a clear topic.

***Author response***

***We are very thankful for the reviewer’s critical read of our manuscript and highlighting of major issues related to its readability. We not only followed the reviewer’s explicit suggestions, but also carefully investigated the language of our manuscript and implemented a large number of improvements to its clarity and readability.***

Reviewer #2 comment #1

Major comments: Line 219. I notice that the subject ID's consist of two letters, and want to double check with the authors to make absolutely sure that the subject ID's do not contain any identifiable or protected health information about the study participants.

***Author response***

***We highly appreciate the attention of the reviewer to this important topic and wish to assure that there is no identifiable nor protected health information disclosed by the subject IDs.***

***As we have mentioned in our Methods section, the subjects of this study are three married couples (marked as couples “A”, “B”, and “C”), and an additional individual (marked as “D”). IDs are composed of two letters where the first indicates the couple affiliation and the second marking the sex (“M” – male, “F” – female). To collect additional samples for long-read sequencing we recruited an additional individual, who is also mentioned in our methods section with a single letter that does not represent any personal information.***

Reviewer #2 comment #2

Line 218-231, 281-285. The authors have done well to both point out a general rule (TM7 populations were exclusive to plaque or tongue), and discuss an exception to the rule (except for T_C_M_Bin_00022). In areas where the authors discuss a general rule and highlight an exception, the text needs to be cleaned up so that it is clear when authors switch from one topic to another.

***Author response***

***We addressed the issue in lines 218-231 by separating the text into two paragraphs (where in the first paragraph we discuss the general rule and in the second paragraph we discuss an exception), and we solved the issue in lines 281-285 by explicitly mentioning that we are going back to describing the general trends.***

Reviewer #2 comment #3

Line 258-341. This subsection seems to lose its way and becomes unfocused. It would benefit substantially from clear topic sentences, shorter paragraphs, and fewer dependent phrases.

***Author response***

***We thank the reviewer for pointing this out. While the findings and hypotheses described in this section remained unchanged, we have carefully reorganized this section by changing its paragraph structure and revising our narrative to improve clarity and readability.***

Reviewer #2 comment #4

Line 404-405. The analyses presented in the manuscript use several types of clustering: clustering of metagenomes, clustering of homologous genes, and clustering of GCs by presence/absence among the metagenomes. The authors should adopt a clear and consistent language to discuss each type of clustering, and signal clearly when the discussion switches from one to another.

***Author response***

***We thank the reviewer for calling attention to this point and we agree that the use of more specific terms for different types of clustering improves the text. Our revised text uses a more explicit language to describe groups of differentially occurring genes, phylogenomic and pangenomic organization of genomes more clearly.***

Reviewer #2 comment #5

Line 599-671. The paper has such a focus on TM7 that I wonder if it shouldn't be mentioned in the title.

***Author response***

***We considered this suggestion carefully and came to the following conclusion: While TM7 has indeed a significant presence in the study, our work also describes important non-TM7 results. We believe that changing the title to reference TM7 explicitly may diminish the visibility of those key sections. Instead we addressed the reviewer’s point by revising our abstract to more explicitly highlight these other aspects of the study. We have added a new sentence at the end of our abstract that reads: “(…). Our data illuminate niche partitioning of enigmatic members of the oral cavity, including SR1 and GN02, and provide genomes for poorly characterized, yet prevalent members of the oral cavity such as uncultivated Flavobacteriaceae”.***

Reviewer #2 comment #6

Line 681-682. How can other researchers use your important results and new genomes in future work? By carrying out this study, you have added to the data resources available to researchers in this area. Please let us know how we can pick up your data and use it in the next study.

***Author response***

***We thank the reviewer for this suggestion, which gave us an opportunity to carefully consider our contributions especially for those who may wish to follow up on them. We added the following section in our conclusions statement to highlight key genomes we have released that may influence future cultivation efforts and improve future applications of comparative genomics to this biome:***

***“Our application of genome-resolved metagenomics to tongue and plaque metagenomes of seven individuals has provided new genomes for prevalent yet uncultivated members of the microbiome. In addition to making accessible 790 MAGs and their coverages across oral metagenomes in the form of reproducible anvi’o profiles (doi:10.6084/m9.figshare.12217805), our study yields a set of 50 genomes (available via the NCBI accession PRJNA625082) that are of special interest to the oral microbiome community due to their novelty. Some of these genomes can inform future cultivation efforts as they suggest the existence of novel lineages with no cultured representatives within relatively well-studied microbial branches, while others, including those that resolve to TM7, SR1 and GN02, can enrich future comparative genomics and metagenomic read recruitment studies as they increase the known microbial gene pool, diversity, and mobilome of the more enigmatic branches of the human oral cavity”.***

Reviewer #2 comment #7

Minor comments: Line 164. If assemblies from plaque samples recruited more reads overall relative to tongue samples, could this be due to the relative abundance of Streptococcus or other common oral bacteria? I imagine that the presence of several closely related species of Streptococcus could diminish the assembly quality and reduce the total number of reads recruited onto contigs.

***Author response***

***The reviewer is correct. Both our previous studies (including doi:10.1073/pnas.1409644111) and our current data (Figure SI12 in the supplementary information file) show that tongue is richer in Streptococcus and other taxa that form complex populations and affect assembly performance. Our revised manuscript includes this text to highlight factors, including strain mixtures:***

***“Confidently assessing the origins of the remaining short reads is difficult as reconstructing genomes from metagenomes is a challenging task that often leads to incomplete genomic descriptions of complex environments such as the human oral cavity. Factors that influence the MAG recovery include the extent of residual eukaryotic host contamination, the poor assembly of strain mixtures, and mobile genetic elements such as viruses and plasmids which are often difficult to bin.”***

Reviewer #2 comment #8

Line 180. Is there a danger that your criteria for inclusion of metagenomes introduces a bias favoring small genomes?

***Author response***

***We assume that by “inclusion of metagenomes” the reviewer meant “inclusion of genomes” or “inclusion of metagenome-assembled genomes”, and believe that the reviewer is referring to the sentence:***

***“Our strict criteria of inclusion of genomes with at least 18 of the 37 ribosomal proteins that we used for phylogenomics removed 539 genomes from the analysis, including 492 low completion (=70%) MAGs, and 24 genomes from the HOMD.”***

***For purposes of the phylogenomic analysis, our strict inclusion criterion would tend to favor more complete genome assemblies. However, the reviewer’s question is more subtle and offering a confident answer is difficult as it would require a near-complete understanding of these complex systems, which is lacking. Our previous experience with genome-resolved metagenomics would suggest that the inclusion criteria defined by high completion and low contamination of single-copy core genes mostly favor relatively clonal populations that have little shared genomic context with others that cooccur in a given environment, regardless of genome size.***

Reviewer #2 comment #9

Line 193. I appreciate that the authors manually refined the metagenomes to guard against contamination. It would be useful for the reader to know how many contaminated genomes were corrected in this way, and what qualities of the assembly led the authors to make the correction.

***Author response***

***We thank the reviewer for this question which made us go back to our notes and identify instances where our second and third manual refinement did result in a significant improvement of our genomes. As our text previously stated, we manually refined all 857 MAGs, where for each MAG we utilized the coverage across the metagenomes from which it was assembled. We updated the text in the section mentioned by the reviewer (line 193) to mention that during our additional rounds of manual refinement “we noticed and corrected binning errors in approximately 9% of our genomes” utilizing public genomic and metagenomic resources.***

***While the quality of the assembly dramatically influences the initial binning results, in our experience the role it plays during refinement is minimal. We also edited the relevant section of the Methods to further expand on what led to the correction of MAGs and how the correction was done:***

***"For each MAG we have refined, we first identified contigs that showed anomalous coverage patterns across samples compared to the rest of the MAG using the program anvi-refine, and visually inspected the nucleotide-level coverage and gene content of such contigs. If we could not confidently assign coverage variation to the presence of hypervariable genomic islands or prophages with matching taxonomy to the MAG, we conservatively eliminated them with the assumption that their inclusion in the MAG was likely due to binning errors."***

Reviewer #2 comment #10

Line 252-257. Here you have a group of sentences that clearly summarize the results of the subsection. I suggest that this be its own paragraph, beginning with, "The phylogenomic analysis…"

***Author response***

***We agree with the reviewer that the busy paragraph buries the key point. We followed the reviewer’s advice regarding the new paragraph and made other minor edits to ensure the key findings stand out.***

Reviewer #2 comment #11

Line 359-387. This subsection is written as one paragraph, but clearly covers three topics: (1) what you set out to do, (2) what you did for quality control, and (3) what the results were. Your important results are getting lost in the middle of a long paragraph.

***Author response***

***We also addressed this by following the reviewer’s suggestion and separated the subsection to multiple paragraphs.***

Reviewer #2 comment #12

Line 493. "nearly all genomes" -- no reason to be vague here. How many?

***Author response***

***The reviewer makes a good point. By “nearly all genomes” we meant 52 of the 55 genomes. We replaced the vague statement with these numbers in our revised manuscript.***

***We very much appreciate the time the reviewer invested into strengthening our manuscript. Their insightful remarks helped us significantly improve the presentation and structure of our work.***

**Second round of review**

**Reviewer 2**

The authors have addressed all the comments in a satisfactory manner. I thank them for their work an attention to detail.
